# The relationship between obesity and obstructive sleep apnea in four community-based cohorts: an individual participant data meta-analysis of 12,860 adults

**DOI:** 10.1016/j.eclinm.2025.103221

**Published:** 2025-04-23

**Authors:** Neda Esmaeili, Laura Gell, Théo Imler, Mohammadreza Hajipour, Luigi Taranto-Montemurro, Ludovico Messineo, Katie L. Stone, Scott A. Sands, Najib Ayas, John Yee, John Cronin, Raphael Heinzer, Andrew Wellman, Susan Redline, Ali Azarbarzin

**Affiliations:** a Division of Sleep and Circadian Disorders, Brigham and Women's Hospital and Harvard Medical School, Boston, MA, USA; b Apnimed, USA; c Center for Investigation and Research in Sleep and Pulmonary Department, University Hospital of Lausanne, Lausanne University, Lausanne, Switzerland; d Department of Medicine, University of British Columbia, Vancouver, BC, Canada; e Department of Epidemiology and Biostatistics, University of California, San Francisco, CA, USA

**Keywords:** Sleep apnea, Obesity, Apnea-hypopnea index, Community-based study, Non-obese, Overweight

## Abstract

**Background:**

Obesity is a well-established risk factor for obstructive sleep apnea (OSA). We assessed the reciprocal prevalence of obesity and OSA and how it varies by age and sex.

**Methods:**

Following a systematic review through March 27, 2025, the final sample included four community-based cohort studies in the US and Switzerland. OSA severity was quantified using the apnea-hypopnea index (AHI, all apneas plus hypopneas with ≥4% oxygen desaturation/hour). Random effects individual participant data (IPD) meta-analyses estimated prevalences. Logistic regression compared odds of OSA across weight groups.

**Findings:**

Among 12,860 adults (mean ± SD age: 66.6 ± 7.3 years), 7222 (56.2%) had OSA (AHI ≥5 events/h) and 3309 (25.7%) had obesity (BMI ≥30 kg/m^2^). IPD meta-analysis showed 31.5% [95% CI: 16.8–48.5] of individuals with OSA had obesity and 44.4% [36.5–52.5] had overweight status (25 ≤ BMI < 30). Among subgroups of individuals with obesity and overweight, 74.3% [63.8–83.5] and 59.8% [46.5–75.7] had any OSA, respectively. Obesity was higher in females than males with OSA, and in younger (<65 years) vs. older individuals. Odds ratios for OSA in subgroups of individuals with overweight and obesity compared to BMI <25 kg/m^2^ were 2.18 [1.73–2.76] and 4.84 [3.09–6.00], respectively.

**Interpretation:**

Our analyses show that most adults with OSA do not have obesity, with 44.4% having overweight and 23.5% having normal weight or underweight. Obesity was more prevalent among females compared to males and in younger individuals (<65 years) compared to older individuals with OSA. Recognizing OSA is not exclusive to obesity highlights the need for personalized treatment plans.

**Funding:**

10.13039/100009886American Academy of Sleep Medicine, 10.13039/100000050National Heart, Lung, and Blood Institute, and 10.13039/100021055Apnimed.

**Research in context**

**Evidence before this study**

Obesity is a major risk factor for obstructive sleep apnea (OSA), and prior research has demonstrated that weight reduction is an effective intervention. However, not all individuals with OSA have obesity, highlighting the need to better understand the overlap between obesity and OSA. Limitations of previous studies include the absence of large-scale analyses estimating the prevalence of obesity among individuals with OSA, reliance on small samples to estimate OSA prevalence in obesity, inconsistent definitions of clinically significant OSA, and insufficient data on prevalence within specific sex and age subgroups.

**Added value of this study**

Our study represents the largest community-based epidemiological sample to investigate the relationship between obesity and OSA, including variations by age and sex. In an individual participant data meta-analysis of 12,860 middle-aged or older adults across four community-based cohorts in the US and Switzerland, most adults with OSA did not have obesity; 44.4% had overweight and 23.5% had healthy weight or underweight. Obesity was more prevalent among females and younger individuals with OSA compared to males and older individuals.

**Implications of all the available evidence**

While obesity is important to target as a modifiable OSA risk factor, it is also important that screening and treatment approaches address OSA in the individuals with overweight or normal weight.

## Introduction

Obstructive sleep apnea (OSA) is characterized by recurrent partial or complete collapse of the upper airways during sleep.^1^^,^^2^ OSA is associated with increased cardiovascular morbidity and mortality risks,^3^, ^4^, ^5^, ^6^, ^7^ cognitive impairment,^8^ economic burden,^9^^,^^10^ and reduced quality of life.^11^

Obesity is a well-established risk factor for OSA^1^^,^^11^, ^12^, ^13^ and its prevalence has continued to rise over the last decades, particularly in developed nations.^13^^,^^14^ People with obesity are at greater risk of OSA due to fat deposition around tongue^15^ and upper airway^12^^,^^16^ (i.e., resulting in a narrower and more collapsible upper airway than people without obesity^12^^,^^17^) and reduced lung volumes associated with central obesity^12^ possibly leading to increased instability in ventilatory control system (i.e., high loop gain).^18^ Although obesity is a major risk factor for OSA, there is growing evidence to suggest that certain non-anatomical endotypes, including low upper airway dilator muscle responsiveness, low arousal threshold, and ventilatory control instability,^19^ as well as anatomical factors beyond obesity^20^ (e.g., narrow bony structure, upper airway length and increased soft palate dimensions) may play significant roles in the pathophysiology of OSA. Furthermore, longitudinal studies have shown associations between weight gain and OSA severity.^11^^,^^21^ Based on these findings, weight reduction by lifestyle intervention,^22^ bariatric surgery,^23^ and pharmacotherapy^24^ are recommended to reduce the severity of OSA in people with overweight or obesity.^11^^,^^25^^,^^26^ Despite weight loss being an effective intervention,^27^^,^^28^ not all individuals with OSA have obesity and there is a need to better understand the overlap of obesity and OSA. Limitations of the prior literature include use of small samples to estimate the prevalence of OSA in obesity^11^^,^^26^^,^^29^^,^^30^ and use of varying definitions for clinically significant OSA.

The primary objective of this study was to examine the prevalence of obesity (and other weight status groups) in OSA using four well-characterized community-based studies with objective sleep data in the United States and Switzerland. Our main hypothesis is that there is considerable proportion of individuals without obesity in OSA. The second objective was to report the prevalence of OSA across different weight status groups, defined by standard criteria defined by the World Health Organization and the Centers for Disease Control and Prevention. In addition, we hypothesized that the association of OSA and obesity differs by age and sex. Therefore, further analyses assessed 1) the reciprocal prevalence of OSA and excess weight status in subgroups defined by age (≥65 vs. <65 years) and sex (female vs. male) and 2) the association of OSA, defined as an apnea-hypopnea index (AHI) ≥5 event/hour, and the body mass index (BMI), after adjusting for different cofounders and covariates.

## Methods

**Fig. 1:**
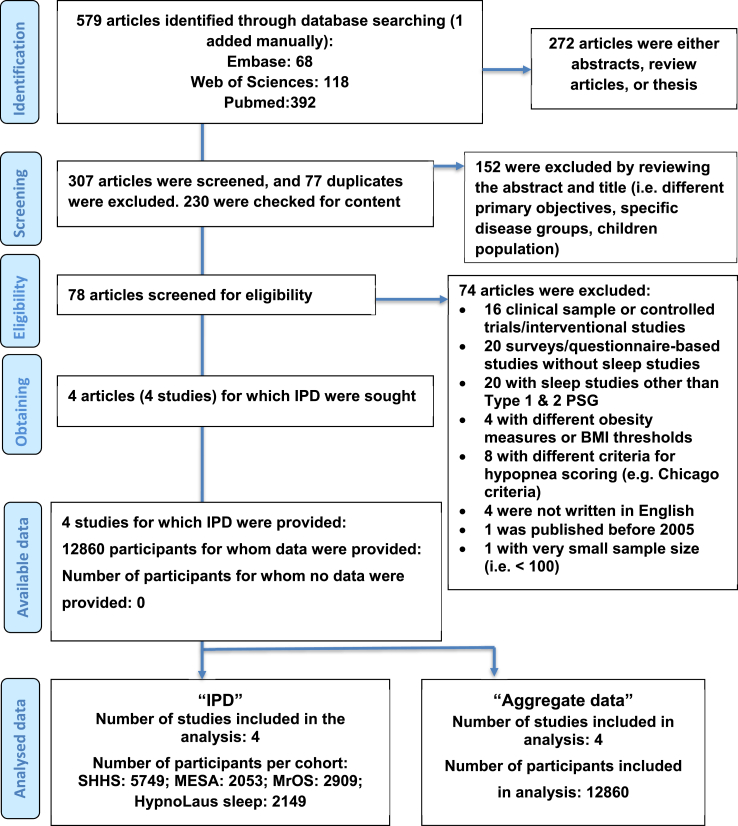
PRISMA IPD flow diagram of study selection.

Study samples were defined through systematic review based on PRISMA IPD guidelines^31^ (Fig. 1) explained in online supplement. Study samples. The sample included the Sleep Heart Health Study (SHHS), the Multi-Ethnic Study of Atherosclerosis (MESA), and the Osteoporotic Fractures in Men Study (MrOS) in the United States (followed similar methods for sleep assessment and the sleep data were scored manually by the certified sleep specialists from the same research group led by SR^32^) and one study in Switzerland (HypnoLaus sleep study; scored by trained sleep technicians and revision of an expert sleep clinician^33^). Details of these studies are shown in the Online Supplement. In all studies, ethical approval was obtained from the local institutional review boards, and all participants provided informed consent. The number of missing variables in the pooled analyses was very low (i.e., <0.5%) and the missing values were considered to occur randomly.

### SHHS

The SHHS study,^32^^,^^34^ included 6441 men and women ≥40 years of age, who completed a standardized questionnaire and underwent a type 2 in–home polysomnography (PSG) between 1995 and 1998 in the first phase of the study. Among 5792 participants on the National Sleep Research Resource (NSRR; sleepdata.org), 5749 individuals (i.e., 99.3% of participants) had required variables for this study.

### MESA

In MESA, of 6814 volunteers from four race and ethnic groups, ages 45 to 84, approximately one-third of the participants were enrolled for sleep assessment in an ancillary study (at examination 5 between 2010 and 2013) that included overnight in–home PSG and sleep questionnaires. Of 2237 individuals invited to undergo sleep evaluations, 2053 had technically acceptable sleep studies^35^ and were included in the current analysis.

### MrOS

In MrOS, of 5994 men ≥65 years of age,^36^^,^^37^ 3135 males took part in the ancillary MrOS Sleep Study from 2003 to 2005, and 2911 had in–home PSG (available on NSRR), as previously described.^38^ Out of 2911 individuals, 2909 people (i.e., 99.9% of participants) had complete data and were included in the analysis.

### HypnoLaus

The HypnoLaus study participants were part of the CoLaus/PsyCoLaus cohort study, described previously.^33^^,^^39^ Of 6733 people aged 35–75 years (from the city of Lausanne, Switzerland), a total of 3043 participants were invited for the sleep study of HypnoLaus (between 2009 and 2013), including in–home PSG and demographic data collection.^33^ Out of 2168 individuals who had PSG, a total of 2149 people with available data (i.e., 99.1% of participants) were included in this study.

### Definition of OSA and obesity

The severity of OSA was based on the AHI, defined as the average number of “apneas” (cessation or near cessation of airflow) plus “hypopneas” (partial reduction in airflow) per hour of sleep. In this study, the hypopneas were defined based on the Centers for Medicare & Medicaid Services (CMS) guidelines (i.e., at least a 30% reduction in thoracoabdominal movement or airflow as compared to baseline, for ≥10 s, with at least 4% oxygen desaturation per hour)^40^ in all four studies.

OSA was identified based on an AHI ≥ 5 events/h. The severity of OSA was characterized using standard thresholds^41^: ≥5–15 events/h (mild OSA), ≥15–30 events/h (moderate OSA), and ≥30 events/h (severe OSA). Weight status was categorized by BMI using the following thresholds: underweight/normal weight (<25 kg/m^2^), overweight (25–30 kg/m^2^) and obese (≥30 kg/m^2^).

#### Statistical analysis

Baseline characteristics are summarized as mean (standard deviation) for numerical variables and as frequency (percentage) for categorical variables.

### Prevalence of weight status groups in OSA

For each study, the proportion of each weight status group (i.e., underweight/normal weight, overweight, and obese) in OSA and its subgroups (mild, moderate, and severe) were determined. After simple pooling of all individual participant data (IPD), these proportions were displayed using Venn diagrams. Then, the weighted pooled prevalence of obesity in OSA and its severity subgroups were estimated by the random effects meta-analysis^42^^,^^43^ to account for between-study variability. Normality assumptions for study effects were verified and the 95% confidence intervals (CIs) for the individual study proportions were computed using the Agresti-Coull (adjusted Wald) method.^44^ Weighted individual study proportions were combined using the inverse variance method after the Arcsine transformation.^45^ The modified Knapp-Hartung method was used to calculate CIs and test statistics for random-effect estimates.^46^^,^^47^ Restricted maximum likelihood (REML) method was used to estimate the between study variances (*τ*^2^).^45^^,^^46^ The studies were also assessed for heterogeneity using the I^2^ test (formulated based on Cochran's Q statistic).^48^ Results were presented on the original probability scale after using the corresponding back-transformation for each study and pooled results. The forest plots were created to graphically display the prevalence estimates and heterogeneity.

### Prevalence of OSA within weight status groups

The proportion of individuals with OSA (overall and across OSA severity subgroups) across weight groups were determined for each study and random effects meta-analyses were used to estimate the pooled prevalence of OSA within weight groups (see above for additional details).

### Prevalence within sex- and age-specific subgroups

The analyses described in the previous two sections were repeated in sex (female vs. male) and age (≥65 vs. <65 years) specific subgroups.

### Association of OSA and BMI categories

The logistic regression models were used to assess the relationship between the any OSA (AHI > 5 events/h) as the dependent variable and BMI categories in each cohort while interaction effect of BMI categories and sex- and age-specific subgroups were assessed in these models. A random-effects IPD meta-analysis (two-stage approach) based on REML was used to estimate the weighted pooled odds ratio between the OSA and BMI categories after adjusting for cofounders (i.e., age, sex, and race), identified based on the modified disjunctive cause criterion^49^(eTable 1 in the online supplement). Logistic regression model assumptions were checked to ensure that the assumptions were not violated.^50^, ^51^, ^52^

### Sensitivity analysis

In additional sensitivity analyses, the meta prevalences were estimated by leaving out one study at a time. Further sensitivity analyses evaluated the associations of OSA and BMI categories after adjusting for additional confounders, identified based on prior studies.^50^, ^51^, ^52^

All statistical analyses were performed using the R statistical package (R Foundation for Statistical Computing; http://www.rproject.org) and p-value <0.05 was considered statistically significant.

### Role of funding

The funding source had no role in the study design, data collection, data analysis, data interpretation, or drafting of the manuscript.

## Results

Summary characteristics of participants across AHI categories for all studies are summarized in Table 1. Overall, the sample included 12,860 participants, of which 7222 (56.2%) were classified with OSA and 3309 (25.7%) with obesity.

**Table 1:** Participants’ characteristics in different OSA severity subgroups.

	N	Age, year	Male (%)	BMI, kg/m^2^	AHI, events/h
AHI < 5 events/h					
SHHS	2799	61 (11)	992 (35.4%)	26.8 (4.5)	2.0 (1.4)
MESA	709	67 (9)	232 (32.7%)	26.7 (5.0)	2.2 (1.5)
MrOS	987	76 (5)	987 (100%)	26.2 (3.4)	2.3 (1.5)
HypnoLaus	1143	56 (11)	428 (37.4%)	24.8 (3.9)	1.8 (1.4)
AHI ≥ 5 events/h					
SHHS	2950	65 (11)	1752 (59.4%)	29.4 (5.3)	18.0 (15.3)
MESA	1344	69 (9)	720 (53.6%)	29.7 (5.5)	21.5 (17.2)
MrOS	1922	77 (5)	1922 (100%)	27.7 (3.9)	19.7 (14.5)
HypnoLaus	1006	62 (11)	618 (61.4%)	27.8 (4.4)	17.5 (15.2)
5 ≤ AHI < 15 events/h					
SHHS	1729	65 (11)	928 (53.7%)	28.8 (5.0)	9.1 (2.9)
MESA	652	69 (9)	289 (44.3%)	28.8 (5.0)	9.3 (2.9)
MrOS	977	76 (5)	977 (100%)	27.2 (3.6)	9.5 (2.8)
HypnoLaus	603	61 (11)	340 (56.4%)	27.0 (4.1)	8.7 (2.9)
15 ≤ AHI < 30 events/h					
SHHS	788	66 (10)	518 (65.7%)	29.9 (5.4)	21.0 (4.3)
MESA	386	69 (9)	220 (57.0%)	29.7 (5.5)	20.9 (4.2)
MrOS	581	77 (5)	581 (100%)	27.8 (4.0)	21.3 (4.3)
HypnoLaus	258	64 (10)	164 (63.6%)	28.3 (4.4)	21.0 (4.4)
AHI ≥ 30 events/h					
SHHS	433	66 (11)	306 (70.7%)	31.2 (6.1)	48.3 (16.8)
MESA	306	69 (9)	211 (69.0%)	31.5 (6.0)	48.4 (14.9)
MrOS	364	77 (5)	364 (100%)	28.8 (4.3)	44.5 (12.5)
HypnoLaus	145	66 (10)	114 (78.6%)	30.2 (4.7)	47.7 (16.4)

AHI, apnea-hypopnea index based on all apneas and hypopneas with ≥4% desaturation (events/h); BMI, body mass index (kg/m^2^); SHHS, Sleep Heart Health Study; MrOS, Osteoporotic Fractures in Men Study; MESA, Multi-Ethnic Study of Atherosclerosis; Quantitative variables (Age, BMI and AHI) were reported as mean (SD).

### Prevalence of weight groups in OSA

**Fig. 2:**
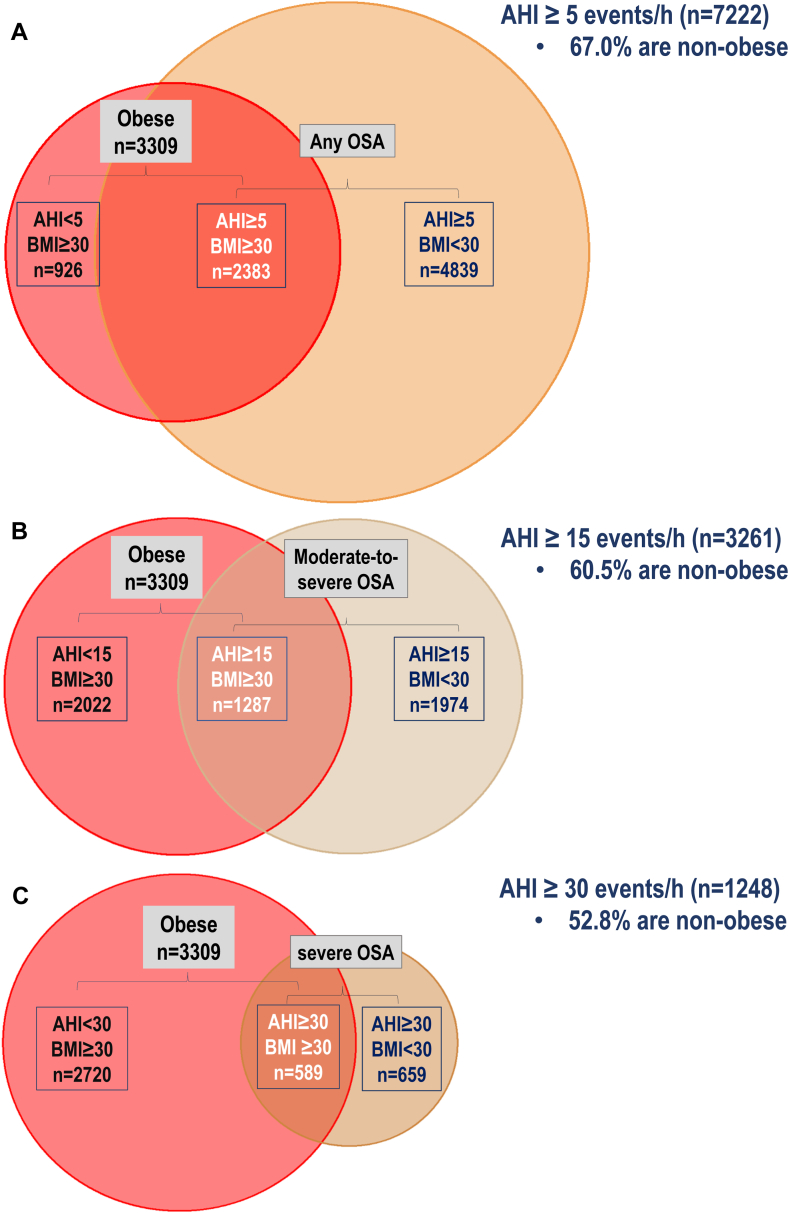
**A**. Intersection of obesity, defined by BMI ≥ 30 kg/m^2^ (red circle) and AHI ≥ 5 events/h (OSA). **B**. Intersection of obesity and AHI ≥ 15 events/h (moderate-to-severe OSA); **C**. Intersection of obesity and AHI ≥ 30 events/h (severe OSA). Percentage of individuals without obesity in each diagram is related to results of random-effects meta-analyses of prevalences.

As shown in Fig. 2, the pooled proportion of individuals with obesity was 33.0% in the group with OSA (AHI ≥5 events/h; N = 7222; Fig. 2A), 39.5% with moderate-to-severe OSA (AHI ≥15 events/h; N = 3261; Fig. 2B), and 47.2% with severe OSA (AHI ≥30 events/h; N = 1248; Fig. 2C).

**Fig. 3:**
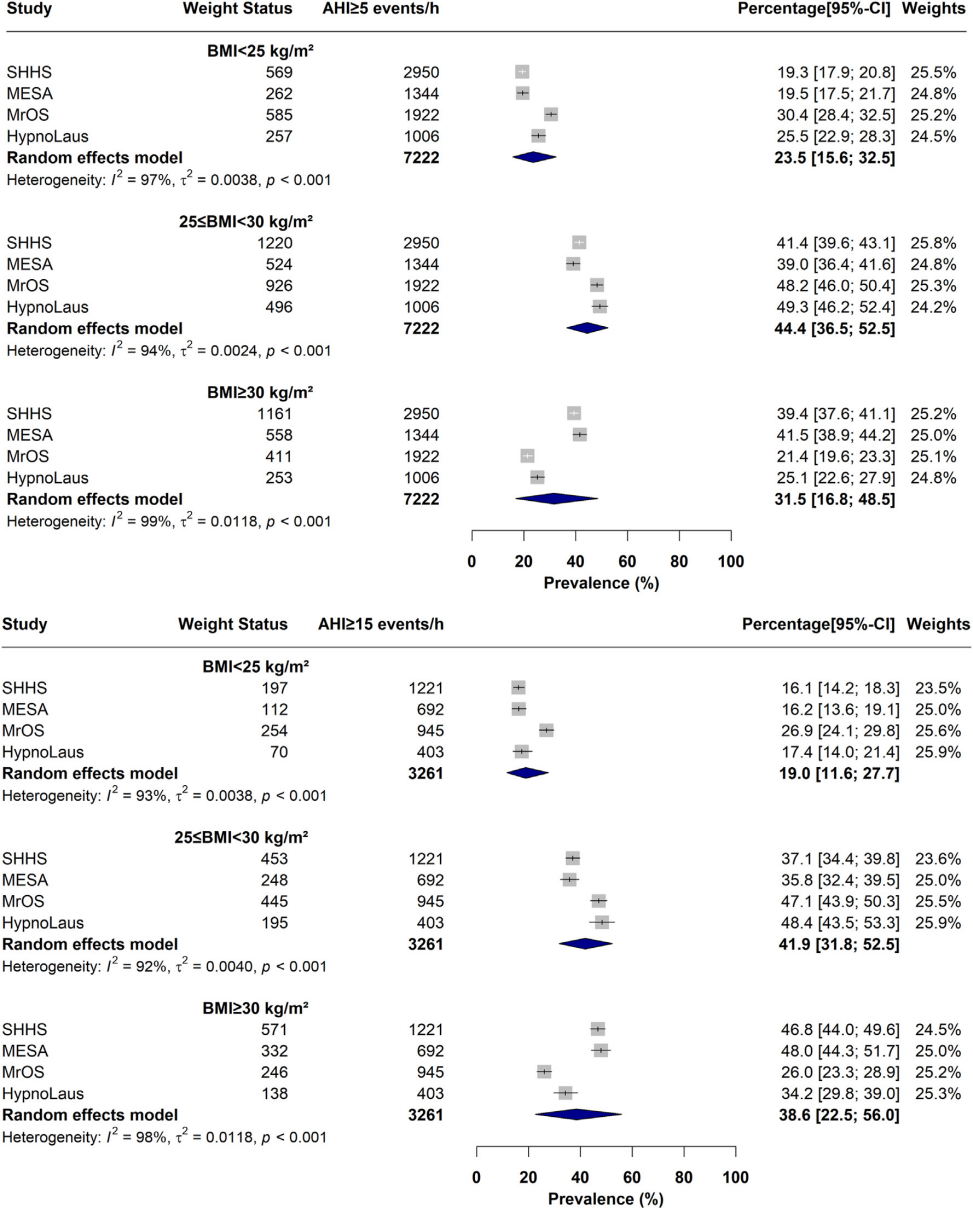
Meta prevalence of different weight groups in OSA, defined as apnea-hypopnea index (AHI) ≥5 events/h and in moderate-to-severe OSA, defined as AHI ≥ 15 events/h. OSA, Obstructive sleep apnea; BMI, body mass index (kg/m^2^); SHHS, Sleep Heart Health Study; MrOS, Osteoporotic Fractures in Men Study; MESA, Multi-Ethnic Study of Atherosclerosis.

Similar prevalences were estimated using random effects IPD meta-analyses after accounting for between-study variability (Fig. 3). In OSA, the pooled prevalences of obesity and overweight status were 31.5 [95% CI: 16.8–48.5]% and 44.4% [36.5–52.5]%, respectively (Fig. 3). In moderate-to-severe OSA, pooled prevalences were 38.6 [22.5–56.0]% for obesity, and 41.9 [31.8–52.5]% for overweight (Fig. 3). Finally in severe OSA, there was a pooled prevalence of 46.7 [29.7–64.1]% for obesity, and 37.3 [24.6–50.9]% for overweight (see eFigures 1–3 for meta-prevalence of weight groups in mild and moderate OSA subgroups, respectively). As shown in eFigure 4, excluding one study at a time did not meaningfully change the meta prevalences of different weight status categories in OSA.

### Prevalence of OSA within weight status groups

**Fig. 4:**
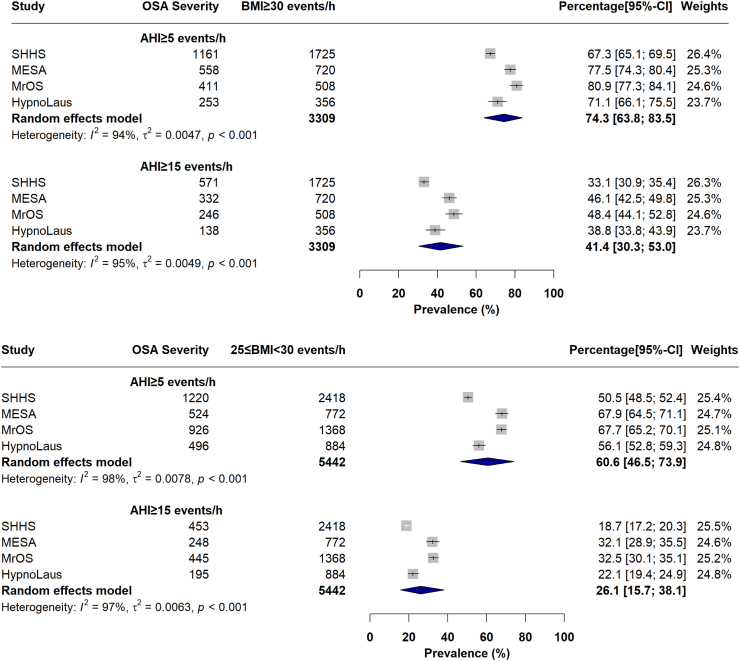
Meta prevalence of OSA, defined as AHI ≥ 5 events/h, and moderate to severe OSA, defined as AHI ≥ 15 events/h, in subgroup of individuals with obesity, defined by BMI ≥ 30 kg/m^2^ (first forest plot) or overweight, defined by 25 ≥ BMI > 30 kg/m^2^ (second forest plot). OSA, Obstructive sleep apnea; AHI, apnea-hypopnea index; BMI, body mass index; SHHS, Sleep Heart Health Study; MrOS, Osteoporotic Fractures in Men Study; MESA, Multi-Ethnic Study of Atherosclerosis.

As shown in Fig. 2, the pooled proportion of individuals with OSA, moderate-to-severe OSA, and severe OSA among participants with obesity (BMI ≥30 kg/m^2^; N = 3309; Fig. 2) was 72.0% (Fig. 2A), 38.9% (Fig. 2B), and 17.8% (Fig. 2C), respectively. The proportion of OSA in the subgroup of individuals with overweight (N = 5442) was 58.2% (eFigure 5). IPD meta-analyses revealed similar prevalences of OSA within subgroups of individuals with obesity and overweight to pooled proportion results (Fig. 4). For example, in the subgroup of individuals with obesity, the estimated pooled prevalence of OSA and moderate-to-severe OSA was 74.3 [95% CI: 63.8–83.5]% and 41.4 [30.3–53.0] %, respectively (Fig. 4). In the subgroup of individuals with overweight, the estimated pooled prevalence of OSA and moderate-to-severe OSA was 60.6 [46.5–73.9]% and 26.1 [15.7–38.1]%, respectively (Fig. 4; See additional eFigures 6–8 for prevalence of OSA categories in different weight status groups). Additionally, meta prevalence rates of OSA within different weight status groups did not change after leaving one study out at a time (See eFigure 9).

### Prevalence within sex- and age-specific subgroups

#### Prevalence of obesity in OSA subgroups

Among men with any OSA (N = 5012), the meta-analysis results showed an obesity prevalence of 28.7 [95% CI: 15.8–43.7]%, while in women with OSA (N = 2210), the obesity prevalence was 40.2 [14.6–69.1]% (eFigure 10). Among older adults with OSA (65 years or older; N = 4837), prevalence of obesity was 28.8 [14.1–46.3]%, while, among younger adults with OSA (<65 years; N = 2385), 40.8 [8.5–78.6]% had obesity (See Online eFigures 10–14 for additional prevalence data in different OSA subgroups).

#### Prevalence of OSA in obesity subgroups

**Fig. 5:**
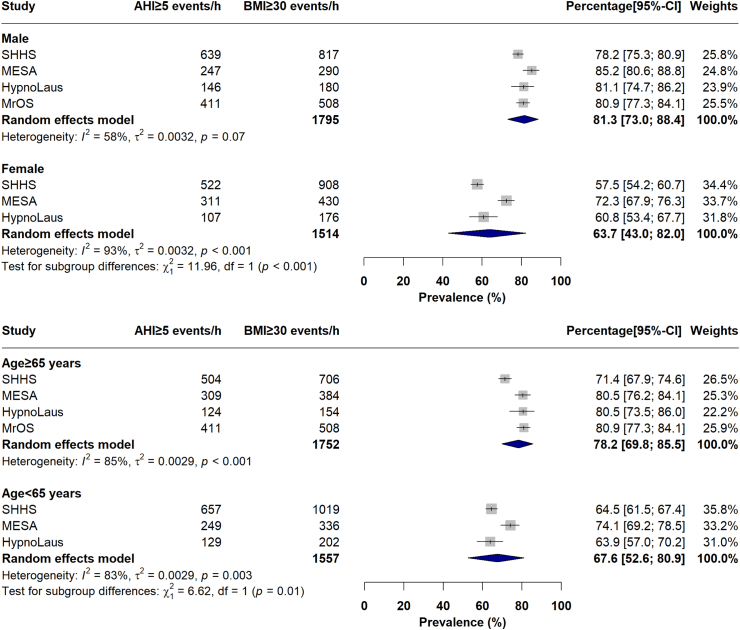
Sex vs. age-specific meta prevalence of OSA, defined as AHI ≥ 5 events/h, in group of individuals with obesity, defined as BMI ≥ 30 kg/m^2^. OSA, Obstructive sleep apnea; AHI, apnea-hypopnea index; BMI, body mass index; SHHS, Sleep Heart Health Study; MrOS, Osteoporotic Fractures in Men Study; MESA, Multi-Ethnic Study of Atherosclerosis.

Sex-specific subgroup meta-analyses estimated the pooled overall OSA prevalence of 81.3 [73.0–88.4]% in the male subgroup with obesity (N = 1795) vs. 63.7 [43.0–82.0]% in the female subgroup with obesity (N = 1514) (Fig. 5). Finally, overall OSA prevalence was estimated to be 78.2 [69.8–85.5]% and 67.6 [52.6–80.9]% in the older subgroup (N = 1752) and younger subgroup with obesity (N = 1557) (Fig. 5, See Online eFigures 14–17).

#### Association of OSA and BMI categories

The pooled odds ratio of having OSA in sex or age–specific subgroups of individuals with overweight and obesity compared to those with underweight/normal weight were shown in eTables 2 and 3.

Finally, the results of sensitivity analyses shown in eTable 4, indicate that incremental adjustment for several confounders did not meaningfully alter the association between BMI categories and OSA.

## Discussion

In a large community-based pooled sample of 12,860 middle-aged or older adults, including three cohorts in the US and one cohort in Switzerland, we show that the majority of individuals with OSA do not have obesity (IPD meta-analysis estimate of 68.5%, Fig. 3) while 23.5% have a BMI < 25.0 kg/m^2^. This observation persisted after restricting the sample to more severe subgroups of OSA, such that in moderate-to-severe OSA, 61.4% are without obesity (19.0% have a BMI < 25.0 kg/m^2^, Fig. 3), and in severe OSA, 53.3% of people are without obesity (15.6% have a BMI <25.0 kg/m^2^, eFigure 1). Consistent with prior literature, this study estimates a high prevalence of OSA in individuals with overweight (60.6%) and obesity (74.3%).^53^^,^^54^ Additionally, an IPD meta-analysis of adjusted odds ratios, revealed that people with obesity have more than 4 times higher odds of having OSA compared to people with a BMI < 25 kg/m^2^. The findings did not meaningfully change after adjusting for additional confounders (See eTable 4).

Excess weight is a major risk factor for the development and progression of OSA.^11^ Several mechanisms may contribute to this relationship, including increased upper airway fat deposition that leads to airway narrowing or collapse during sleep,^55^, ^56^, ^57^ changes in respiratory mechanics,^12^^,^^58^ impaired ventilatory control system,^59^ alteration in secretion of hormonal (e.g., leptin,^12^ adiponectin^12^) and inflammatory factors by adipose tissues (e.g.,TNF-alpha^12^) which may contribute to the pathogenesis of OSA. On the other hand, previous studies have shown that OSA may increase the risk of obesity through mechanisms including OSA-related hormonal dysregulation,^60^ decreased physical activity,^25^ metabolic disturbances such as insulin resistance and dyslipidemia,^61^ promoting weight gain, increased caloric intake,^62^ and elevated levels of pro-inflammatory cytokines, potentially contributing to metabolic dysfunction^59^ and weight gain. Despite these associations, CPAP treatment does not appear to result in weight loss.^63^^,^^64^ While these studies highlight complex relationships between OSA and obesity, emphasizing the importance of managing both conditions, our findings show that OSA can affect individuals of various body compositions in general population and the diagnosis and treatment of OSA should be based on comprehensive evaluation and consideration of multiple factors beyond weight alone.

Previous studies have estimated that 9–38% of general population has OSA, defined by an AHI ≥ 5 events/h.^65^ These estimates increase by age, male sex, and obesity.^66^ In adults with obesity, for example, the prevalence of OSA was nearly double or higher than those with normal weight.^21^^,^^67^ Consistent with these findings, we demonstrated that about 74% of adults with obesity aged 35–90 years have OSA (defined as AHI ≥5 events/h), while for a more clinically significant OSA (AHI ≥15 events/h), the prevalence rate is about 41% (Fig. 4).

Our study provides further information on the prevalence of obesity and overweight status in OSA, which has not been studied extensively in the past.^68^ Our findings revealed that the majority of individuals with OSA do not have obesity (meta-analysis estimate of 44.4% overweight and 23.5% healthy weight/underweight; Fig. 3). The overall prevalence of overweight/obesity is notably consistent with prior literature.^69^

Our findings also show that, in participants with OSA, a higher proportion of females (vs. males) and younger adults (vs. older adults) have obesity, suggesting that obesity may be a stronger risk factor for OSA in younger or female adults^13^

However, due to the rising prevalence of obesity in the general population^70^^,^^71^ since these studies were conducted, we advise caution when extrapolating these findings to the present day. Furthermore, a higher prevalence of obesity may be observed in a clinical sample when people present with symptoms and other comorbidities.^72^^,^^73^ Nonetheless, these findings suggest that anatomical or pathophysiological factors other than obesity may play a more significant role in the development of OSA than previously believed. For instance, the upper airway narrowing in OSA could stem from a small bony structure where soft tissue, other than fat, could diminish airway size. The redistribution of fluids towards the neck when individuals lie down in bed^74^ is a recognized factor that leads to increased resistance in the upper airway, along with the increased size of pharyngeal lymphoid tissue^75^ or a larger uvula,^76^ both linked to heightened collapsibility of the upper airway independently of obesity. Similarly non-anatomical causes of OSA, such as ventilatory instability (i.e., elevated loop gain), diminished compensatory capacity of upper airway muscles, and a low arousal threshold may play a more significant role in OSA development than previously assumed.^19^ Finally, as expected, our findings show that, there is a higher proportion of OSA in males (vs. females) and older (vs. younger) participants with obesity.

The high prevalence of normal weight people in OSA carries significant public health implications. First, underdiagnosis/misdiagnosis of OSA in individuals with a BMI < 25 kg/m^2^ may not be promptly recognized due to the common misperception that OSA is primarily associated with obesity. Second, OSA has been shown to be associated with various cardiovascular diseases (CVDs),^77^ such as hypertension,^60^ arrhythmias,^78^ and stroke^78^^,^^79^ independent of obesity. Therefore, normal weight individuals with untreated OSA may be at increased risk of CVD. Third, in addition to CVD, untreated OSA in people with a BMI < 25 kg/m^2^ is associated with excessive daytime sleepiness,^8^ impaired cognitive function,^8^ increased risk of car crashes,^80^ and impaired quality of life^8^ as well as work-related injuries.^81^ Therefore, public health efforts should focus on increasing awareness about the diverse risk factors and presentations of OSA, beyond obesity.

In people with obesity and moderate-to-severe OSA, weight loss treatment with tirzepatide (SURMONT-OSA trial), a dual glucagon-like peptide-1 (GLP-1) and glucose-dependent insulinotropic polypeptide (GIP) receptor agonist, has been shown to reduce the AHI by 48–56%, resulting in a meaningful improvement in systolic blood pressure and patient reported outcomes.^28^ While this provides a promising avenue for pharmacologic treatment of OSA, our findings show that only 17.8% of individuals with any OSA would have met the inclusion criteria of SURMONT-OSA trial (AHI ≥ 15 events/h and BMI ≥ 30 kg/m^2^). To maximize public health benefits, a balanced approach is essential. This should include promoting weight loss where appropriate, while also ensuring that non-obesity related factors are thoroughly investigated and addressed in OSA management.

This study has several strengths and limitations. First, to the best of our knowledge, this study is the largest community-based epidemiological sample to examine the prevalence of obesity and excess weight in OSA and its subgroups (eTable 5). Using the same definitions and methods in all steps, we used three well-characterized cohorts in the US and one in Switzerland to extract the true proportions and estimate the prevalence rates using robust statistical analysis methods.^66^ Second, all these studies used type I or type II polysomnography devices to quantify the AHI and the sleep studies were scored consistently across studies (SHHS, MESA and MrOS studies were scored by same team). Third, the presence of OSA was defined consistently across all studies as an AHI ≥5 events/h in which the included hypopneas were associated with ≥4% desaturation. Based on this definition of AHI, severe OSA was shown to be associated with increased risk of mortality^7^ and car crashes.^80^ Fourth, robust statistical methods were used to account for potential between-study variability and to provide a more precise estimate of prevalence rates. However, our study has important limitations, including 1) inadequate presentation of all racial and ethnic groups; 2) the mean age of all participants ranged from 57.2 years in HypnoLaus cohort to 76.0 years in MrOS, therefore, these findings may not be fully generalizable to younger people with OSA; 3) the SHHS over-recruited symptomatic participants and therefore it may not be a representative sample of the general public; 4) BMI was used to measure obesity and other weight status subgroups. While BMI is accepted as a standard measure of obesity, it may be limited in accurately quantifying the abdominal obesity and fat deposition around neck. These specific measures of obesity (waist or neck circumference) may have a stronger association with OSA and need to be further examined in future epidemiological studies; 5) These samples were collected between 1995 and 2015. However, the prevalence of obesity has been rising since then and the reported rates may not reflect the current prevalence of both OSA and obesity. 6) The interaction between age and BMI categories in association with OSA was reported using the multiplicative scale (odds ratio; eTable 3). However, the additive scales revealed similar findings (data not reported); 7) Cofounders were selected based on the modified disjunctive cause criterion^49^; however, the impact of unmeasured confounding (e.g., the socioeconomic status) warrants further investigation; 8) High I^2^ statistics in our studies are common in meta-analyses of proportions and do not necessarily indicate significant variability between studies.^82^ Indeed, when a small number of studies are available, visual inspection of forest plots and sensitivity analyses (e.g., subgroup analyses) are strongly recommended^82^; and finally, 9) Although the approach used here accounts for study-level variability through inverse-variance weighting in a random-effects model, the absence of inverse probability weighting could potentially indicate that any unmeasured differences in study populations could influence the pooled prevalence estimates. As a result, the pooled prevalences may not represent unbiased estimates for any specific real-world population due to the omission of population weighting.

In four large community-based studies, it was found that a majority of individuals with OSA do not have obesity. While a significant proportion of individuals with OSA have BMI in overweight range (BMI 25–30), 23.5% were with normal weight or underweight. This trend persisted even among those with moderate-to-severe and severe OSA. The study also noted a high prevalence of OSA among individuals with overweight and obesity, with higher BMI correlating with increased OSA severity. The study underscores the importance of tailored OSA treatment approaches that consider individual characteristics in addition to risk factors.

## Contributors

Conception: NE, LG, JY, L T-M, JC, AA. Study design: NE, LG, JY, L T-M, JC, NA, AA. Data analysis: NE, LG, TI, AA. Parent study design and data collection: TI, KS, RH, SR. Statistical analysis: NE, LG, TI, AA. Initial drafting of the manuscript: NE, AA. Current Analytic Funding: NE, AA. NE directly accessed and verified the MESA, MrOS, and SHHS data. TI directly accessed and verified the HypnoLaus data. All authors interpreted data, edited the manuscript for important intellectual content, and approved the final draft.

## Data sharing statement

Deidentified signals, covariates, and outcomes data were obtained under separate collaborative agreement from the parent studies. Individual data presented in the current study can be obtained by request but may require a three-way data use agreement with parent cohort investigators.

## Declaration of interests

TI, MH, LM reports grant fundings from Apnimed unrelated to this work. LG, L T-M, JY, JC work for Apnimed. AW works as a consultant for Apnimed, Nox, Inspire, and Somnifix International LLC. He has received grants from Sanofi and Somnifix. He also has a financial interest in Apnimed Corp., a company developing pharmacologic therapies for sleep apnea. SS reports grant support from Apnimed, Prosomnus, and Dynaflex, and has served as a consultant for Apnimed, Nox Medical, Inspire Medical Systems, Eli Lilly, Respicardia, LinguaFlex, and Achaemenid. He receives royalties for intellectual property pertaining to combination pharmacotherapy for sleep apnea via his Institution. He is also co-inventor of intellectual property pertaining to wearable sleep apnea phenotyping, unrelated to the current manuscript, also via his Institution. NA has received speaker and consultancy fees from Eli Lilly. KS received grant from Eli Lilly to explore barriers and facilitators to screening for OSA in primary care. AW's interests were reviewed and are managed by Brigham and Women's Hospital and Partners HealthCare in accordance with their conflict-of-interest policies. RH has received speaker or consultancy fees from Resmed, Jazz, Inspire, Bioprojet, Philips, Merck, Nyxoah, Medtronic, Nestlé and Löwenstein. AA reports grant support from Somnifix and serves as a consultant for Somnifix, Respicardia, Eli Lilly, Inspire, Cerebra and Apnimed. Apnimed is developing pharmacological treatments for Obstructive Sleep Apnea. AA is also co-inventor of intellectual property pertaining to wearable sleep apnea phenotyping, unrelated to the current manuscript. AA received speaker fees from ProSomnus. AA's interests were reviewed by Brigham and Women's Hospital and Mass General Brigham in accordance with their institutional policies. RH reports grant from Apnimed, Ligue pulmonaire vaudoise, and serves as a consultant for Resmed, Nyxoah, Apnimed-Shionogi and Nomics. SR received consulting fees from Eli Lilly Inc and has been an unpaid member of the Apnimed Scientific Advisory Committee.

## Appendix A Supplementary data

Online Suppliment clean
