# Supplementary material for: The relationship between obesity and obstructive sleep apnea in four community-based cohorts: an individual participant data meta-analysis of 12,860 adults
Source: eClinicalMedicine. 2025 Apr 23;83:103221. doi: 10.1016/j.eclinm.2025.103221 (PMC12051718; doi:10.1016/j.eclinm.2025.103221)
Supplement: Online Suppliment clean [file mmc1.docx]

**ONLINE SUPPLEMENTARY DATA**

**The Relationship Between Obesity and Obstructive Sleep Apnea** **in Four Community-based Cohorts: An Individual Participant Data Meta-Analysis of 12,860 Adults.**

Neda Esmaeili^1^, PhD, Laura Gell^1,2^, PhD, Théo Imler^3^, MS, Mohammadreza Hajipour^4^, MS, Luigi Taranto-Montemurro^1,2^, MD, Ludovico Messineo^1^, MD, Katie L Stone^5^, PhD, Scott A Sands^1^, PhD, Najib Ayas^4^_,_ John Yee^2^, MD, MPH, John Cronin^2^, MD, Raphael Heinzer^3^, MD, Andrew Wellman^1^, MD, PhD, Susan Redline^1^, MD, Ali Azarbarzin^1^, PhD

^1^Division of Sleep and Circadian Disorders, Brigham and Women's Hospital and Harvard Medical School, Boston, MA, USA; ^2^Apnimed; ^3^Center for Investigation and Research in Sleep and Pulmonary Department, University Hospital of Lausanne, Lausanne University, Lausanne, Switzerland; ^4^University of British Columbia, Vancouver, BC, Canada; ^5^Department of Epidemiology and Biostatistics, University of California, San Francisco, CA, USA.

**Study Design**

We designed a systematic review modeled based on current PRISMA (Preferred Reporting Items for Systematic Reviews and Meta-Analyses) IPD statements^1^. The PubMed, Embase, and Web of Science databases were searched for relevant reports. For PubMed, topic-related terms were combined using Boolean operators and the following search was conducted: ((sleep apnea[Title] OR Sleep-Disordered Breathing[Title])) AND ((obesity[Title] OR weight[Title] OR obese[Title] OR fat[Title] OR prevalence[Title])) AND ((adult[Abstract] OR old[Abstract])) AND ((population[Abstract] OR community[Abstract])). For the other two databases, their respective operators were used with the same search terms. Two independent reviewers selected the papers and excluded the abstracts, review papers, duplicates and other unrelated papers by help of review manager software. The titles and abstracts of rest of articles were screened using the eligibility criteria which included main goals of studies, adult community-based population and reported mainly in English; Those for which insufficient information was found in the title and/or abstract, underwent full-text analysis to determine their eligibility and finally any disagreement between reviewers was resolved by consulting with third reviewer (A.A.). See Figure 1 [PRISMA flow diagram] for more information. The PRISMA-IPD checklist was provided.

**Study samples.** We requested the IPD through paper proposals and collaborative agreements. The sample included the Sleep Heart Health Study (SHHS), the Multi-Ethnic Study of Atherosclerosis (MESA), and the Osteoporotic Fractures in Men Study (MrOS) in United States (followed similar methods for sleep assessment and the sleep data were scored by the same research group led by SR^2^) and one study in Switzerland (HypnoLaus sleep study).

In all studies, ethical approval was obtained from the local institutional review boards, and all participants provided informed consent. All studies excluded participants who reported using any kind of therapy, such as mouthpieces, oxygen therapy, bilevel positive airway pressure, or CPAP.

SHHS:

The SHHS cohort is a community-based, multi-center prospective cohort study in the US, designed to investigate the effects of sleep disordered breathing cardiovascular (CV) outcomes. The SHHS study^2,3^, included 6,441 men and women ≥ 40 years of age, who completed a standardized questionnaire and underwent a type 2 polysomnography (PSG) between 1995-1998 in the first phase of the study. Among 5792 participants on the National Sleep Research Resource (sleepdata.org), 5749 individuals had required variables for this study.

MESA:

The MESA study is a prospective, community-based, ethnically diverse cohort study designed to study the prevalence and development of subclinical cardiovascular disorders. In summary, 6,814 volunteers from four race and ethnic groups, ages 45 to 84, were recruited at six centers in the US. Participants were free of clinical CV diseases at the baseline examination (examination 1 in 2000–2002). At examination 5 (2010–2013), approximately one-third of the participants were enrolled for sleep assessment in an ancillary study that included overnight in-home PSG and sleep questionnaires. Of 2,237 individuals invited to undergo sleep evaluations, 2,053 had technically acceptable sleep studies^4^ and were included in the current analysis.

MrOS:

The primary MrOS study is a community-based, prospective cohort study that was conducted between 2000 and 2002 and included 5994 men aged 65 and older who were recruited from six different U.S. sites^5,6^. The goal of the study was to characterize the osteoporosis and fracture epidemiology in older men^7^.

3135 males from the MrOS cohort took part in the ancillary MrOS Sleep Study from 2003 to 2005, where they underwent in-home PSG, as previously described^3^. Out of 3135 individuals, PSGs from 2911 satisfied the preliminary research quality requirements and were made available on the National Sleep Research Resource website (sleepdata.org). For this study, 2909 people had complete data and were included in the analysis.

HypnoLaus:

The HypnoLaus study participants were part of the CoLaus/PsyCoLaus cohort study, described previously^8,9^. The CoLaus/PsyCoLaus study was conducted between 2003 and 2006 and included a sample of 6733 people aged 35–75 years, from the city of Lausanne, Switzerland. The aim of the study was to investigate the prevalence of cardiovascular risk factors and psychiatric disorders in the general population. Between 2009 and 2013, a total of 3043 consecutive participants of the CoLaus/PsyCoLaus study were invited to participate in the population-based sleep study of HypnoLaus, including in-home PSG and demographic data collection^8^. A total of 2149 people with available data were included in this study.

**Power calculations:** The power calculation was done based on the smallest subgroups in HypnoLaus cohort (females with obesity: n=176; and males with obesity: n=180):

For these subgroups, we tested the hypothesis that the prevalence of OSA among females and males with obesity is higher than in females and males in the general population. The estimated prevalence of OSA (AHI ≥ 5 events/hour) in the general population is 30% for females and 45% for males ^10,11^. In our smallest subgroup, the observed prevalence of OSA among females and males with obesity was 61% and 81%, respectively, resulting in Cohen’s *g* effect sizes of 0.31 and 0.36. Exact tests for proportions (Difference from Constant, Binomial One-Sample Test) indicated a power of 100% for each subgroup (α = 0.05, two-tailed).

**References:**

1. Stewart, L. A. *et al.* Preferred reporting items for a systematic review and meta-analysis of individual participant data: the PRISMA-IPD statement. *JAMA* **313**, 1657–1665 (2015).

2. Redline, S. *et al.* Methods for obtaining and analyzing unattended polysomnography data for a multicenter study. *Sleep* **21**, (1998).

3. Quan, S. F. *et al.* The sleep heart health study: design, rationale, and methods. *Sleep* **20**, 1077–1085 (1997).

4. Geovanini, G. R. *et al.* Association between obstructive sleep apnea and cardiovascular risk factors: variation by age, sex, and race. The Multi-Ethnic Study of Atherosclerosis. *Ann Am Thorac Soc* **15**, 970–977 (2018).

5. Blank, J. B. *et al.* Overview of recruitment for the osteoporotic fractures in men study (MrOS). *Contemp Clin Trials* **26**, 557–568 (2005).

6. The Osteoporotic Fractures in Men (MrOS) Study. https://mrosonline.ucsf.edu  . *National Institutes of Health*.

7. Orwoll, E. *et al.* Design and baseline characteristics of the osteoporotic fractures in men (MrOS) study—a large observational study of the determinants of fracture in older men. *Contemp Clin Trials* **26**, 569–585 (2005).

8. Heinzer, R. *et al.* Prevalence of sleep-disordered breathing in the general population: the HypnoLaus study. *Lancet Respir Med* **3**, 310–318 (2015).

9. Firmann, M. *et al.* The CoLaus study: a population-based study to investigate the epidemiology and genetic determinants of cardiovascular risk factors and metabolic syndrome. *BMC Cardiovasc Disord* **8**, 1–11 (2008).

10. Senaratna, C. V *et al.* Prevalence of obstructive sleep apnea in the general population: a systematic review. *Sleep Med Rev* **34**, 70–81 (2017).

11. Tufik, S., Santos-Silva, R., Taddei, J. A. & Bittencourt, L. R. A. Obstructive sleep apnea syndrome in the Sao Paulo epidemiologic sleep study. *Sleep Med* **11**, 441–446 (2010).

| **eTable 1: Modified disjunctive cause criterion confounder selection** | | | | |
| --- | --- | --- | --- | --- |
| **Covariate** | **Cause of obesity** | **Cause of OSA** | **Notes** | **Keep in model?** |
| Age | ✓ | ✓ | Common cause | ✓ |
| Sex | ✓ | ✓ | Common cause | ✓ |
| Race/Ethnicity | ✓ | ✓ | Common cause | ✓ |
| Socioeconomic Status | ✓ | ✓ | Common cause | ✓ |
| Smoking | X | ✓ | OSA risk factor | ✓ |
| Alcohol Use | ✓ | ✓ | Common cause | ✓ |
| Physical Activity | ✓ | X/✓ | Causes BMI; possibly affects OSA indirectly | ✓ |
| Prevalent CVD | X | ✓ | Associated with OSA | ✓ |
| Hypertension | X | ✓ | Associated with OSA | ✓ |
| Diabetes Mellitus | X | ✓ | Associated with OSA | ✓ |
| Neck Circumference | ✓ | ✓ | Likely a mediator or proxy of BMI | X |

| **eTable 2: Logistic regression models showing the association of BMI categories with OSA in age subgroups.** | | | | |
| --- | --- | --- | --- | --- |
| **Comparison** | **Study** | **Odds Ratio [95% CI]** | | **Interaction Odds Ratio [95% CI]** |
|  |  | **Age < 65 years** | **Age ≥ 65 years** |  |
| BMI: 25-30 vs. BMI <25 kg/m^2^ | SHHS | 2.01 [1.64 - 2.47]*** | 1.41 [1.17-1.71]*** | 0.70[0.53 - 0.93], **p = 0.01** |
|  | MrOS |  | 1.66 [1.40 - 1.97]*** |  |
|  | MESA | 3.57 [2.39 - 5.36]*** | 2.26 [1.67 - 3.08]*** | 0.64 [0.39 - 1.04], p = 0.07 |
|  | HypnoLaus | 2.59 [2.00 - 3.36]*** | 2.81 [1.96 - 4.06]*** | 1.09 [0.70 - 1.70], p = 0.72 |
|  | Pooled Estimate | 2.55 [1.87 - 3.47]***  AIC:4.6 | 1.91 [1.42 - 2.55]***  AIC: 5.3 | 0.77 [0.58 - 1.02], p = 0.08  ꚍ^2^ = 0.0; I^2^ = 35.6%; AIC: 4.4 |
| BMI: ≥30 vs. BMI <25 kg/m^2^ | SHHS | 5.68 [4.60 - 7.04]*** | 1.94[1.42 - 2.64]*** | 0.52[0.38 - 0.70]*** |
|  | MrOS |  | 3.43 [2.66 - 4.46]*** |  |
|  | MESA | 8.97 [5.95 - 13.68]*** | 4.75 [3.34 - 6.82]*** | 0.53 [0.31 - 0.89], p = 0.02 |
|  | HypnoLaus | 6.06 [4.24 - 8.72]*** | 4.96 [3.09 - 8.14]*** | 0.82 [0.45 - 1.51], p = 0.51 |
|  | Pooled Estimate | 6.45 [ 5.04 - 8.27]***  AIC:3.9 | 3.73 [2.92 - 4.77]***  AIC: 4.3 | 0.56 [0.44 - 0.71]***  ꚍ^2^ = 0.0; I^2^ = 0.0%; AIC: 3.9 |
| SHHS: Sleep Heart Health Study. MrOS: Osteoporotic Fractures in Men Study. MESA: Multi-Ethnic Study of Atherosclerosis. Pooled effect was estimated using random-effect meta-analysis of regression coefficients. p-value <0.001: *** . Confounders were sex, race, age. AIC: Akaike Information Criterion;  Sensitivity analyses were done by leave-one-out-method for pooled estimates. | | | | |

| **eTable 3: Logistic regression models showing the association of BMI categories with OSA in sex subgroups.** | | | | |
| --- | --- | --- | --- | --- |
| **Comparison** | **Study** | **Odds Ratio [95% CI]** | | **Interaction Odds Ratio [95% CI], p-value** |
|  |  | **Female** | **Male** |  |
| BMI: 25-30 vs. BMI <25 kg/m^2^ | SHHS | 1.64 [1.35 - 1.98]*** | 1.69 [1.38 - 2.07]*** | 1.03 [0.78 - 1.36], p = 0.82 |
|  | MrOS |  | 1.66 [1.40 - 1.97]*** |  |
|  | MESA | 2.84 [2.05 - 3.95]*** | 2.47 [1.72 - 3.54]*** | 0.87 [0.54 - 1.40], p = 0.57 |
|  | HypnoLaus | 2.43 [1.80 - 3.29]*** | 2.91 [2.16 - 3.93]*** | 1.20 [0.78 - 1.83], p = 0.40 |
|  | Pooled Estimate | 2.19 [1.57 - 3.07]***  AIC: 4.7 | 2.06 [1.57 - 2.72]***  AIC: 4.9 | 1.04 [0.84 -1.28], p = 0.74  ꚍ^2^ = 0.0; I^2^ = 0.0%; AIC: 2.2 |
| BMI: ≥30 vs. BMI <25 kg/m^2^ | SHHS | 4.08 [3.35 - 4.99]*** | 4.36 [3.44- 5.53]*** | 1.07 [0.78 - 1.45], p = 0.68 |
|  | MrOS |  | 3.43 [2.66 - 4.46]*** |  |
|  | MESA | 6.94 [4.94 - 9.81]*** | 5.12 [3.33 - 7.98]*** | 0.74 [ 0.54 - 1.40], p = 0.26 |
|  | HypnoLaus | 5.14 [3.52 - 7.56]*** | 6.37 [4.10 - 10.10]*** | 1.24 [0.69 - 2.25], p = 0.48 |
|  | Pooled Estimate | 5.14 [ 3.73 -7.06]***  AIC: 4.5 | 4.47 [3.50 - 5.71]***  AIC: 4.4 | 1.01 [0.79 – 1.29], p = 0.92  ꚍ^2^ = 0.0; I^2^ = 0.0%; AIC: 4.0 |
| SHHS: Sleep Heart Health Study. MrOS: Osteoporotic Fractures in Men Study. MESA: Multi-Ethnic Study of Atherosclerosis. Pooled effect was estimated using random-effect meta-analysis of regression coefficients; AIC: Akaike Information Criterion; *** : p-value <0.001; Confounders were race, and age. | | | | |

| **eTable 4. Logistic regression models showing the association of BMI categories with OSA after additional adjustment for covariates.** | | | | |
| --- | --- | --- | --- | --- |
|  | | **Model 1**  **Odds ratio [95%CI]** | **Model 2**  **Odds ratio [95%CI]** | **Model 3**  **Odds ratio [95%CI]** |
| BMI: 25-30 vs. BMI <25 kg/m^2^ | SHHS | 1.66 [1.45-1.91]*** | 1.58 [1.36-1.83]*** | 1.58 [1.35-1.86]*** |
|  | MrOS | 1.66 [1.40-1.97]*** | 1.70 [1.43-2.02]*** | 1.68 [1.40-2.01]*** |
|  | MESA | 2.60 [1.99-3.40]*** | 2.59 [1.98-3.39]*** | 2.57 [1.96-3.37]*** |
|  | **Pooled Estimate** | **1.89 [1.23-2.91]***** | **1.87 [1.19-2.94]***** | **1.86 [1.20-2.91]***** |
| BMI ≥30 vs. BMI <25 kg/m^2^ | SHHS | 4.19 [3.60-4.89]*** | 3.92 [3.31-4.64]*** | 3.92 [3.26-4.71]*** |
|  | MrOS | 3.43 [2.65-4.44]*** | 3.55 [2.73-4.61]*** | 3.41 [2.58-4.50]*** |
|  | MESA | 6.68 [4.90-9.09]*** | 6.60 [4.83-9.01]*** | 6.33 [4.61-8.70]*** |
|  | **Pooled Estimate** | **4.53 [2.52-8.12]***** | **4.45 [2.52-7.84]***** | **4.34 [2.52-7.46]***** |
| *** : p-value < 0.001;  Model1: age, sex, race.  Model2: Model1+ smoking status, alcohol consumption, and vigorous physical activity level.  Model3: Model2 + prevalent CVD, Hypertension, Diabetes Mellitus. | | | | |

| **eTable 5.** **Participants’ characteristics in each cohort.** | | | | |
| --- | --- | --- | --- | --- |
|  | **SHHS** | **MESA** | **MrOS** | **HypnoLaus** |
| Sample size, n | 5749 | 2053 | 2909 | 2149 |
| Age, years | 63 (11) | 68 (9) | 76 (5) | 59 (11) |
| Male sex, n (%) | 2744 (47.7%) | 952 (46.4%) | 2909 (100%) | 1046 (48.7%) |
| BMI, kg/m^2^ | 28.2 (5.1) | 28.7 (5.5) | 27.2 (3.8) | 26.2 (4.4) |
| AHI, events/h | 10.2 (13.6) | 14.8 (16.7) | 13.8 (14.4) | 9.1 (13.0) |
| Race, n (%) |  |  |  |  |
| White | 4880 (84.9%) | 741 (36.1%) | 2639 (90.7%) | 1995 (92.8%) |
| Black | 496 (8.6%) | - | - | 44 (2.1%) |
| African American | - | 572 (27.9%) | 99 (3.4%) | - |
| Asian | - | - | 84 (2.9%) | 23 (1.1%) |
| Chinese American | - | 250 (12.2%) | - | - |
| Hispanic | - | 490 (23.8%) | 55 (1.9%) | 39 (1.8%) |
| Other | 373 (6.5%) | - | 32 (1.1%) | 48 (2.2%) |
| BMI: body mass index, AHI: Apnea-Hypopnea Index, SHHS: Sleep Heart Health Study. MrOS: Osteoporotic Fractures in Men Study. MESA: Multi-Ethnic Study of Atherosclerosis. Quantitative variables (Age, BMI and AHI) were reported as mean (SD). | | | | |

**eFigure 1. Meta prevalence of different weight groups in severe OSA, defined as AHI≥ 30 events/h. OSA: Obstructive sleep apnea. AHI: apnea-hypopnea index. BMI: body mass index (kg/m^2^). SHHS: Sleep Heart Health Study. MrOS: Osteoporotic Fractures in Men Study. MESA: Multi-Ethnic Study of Atherosclerosis**


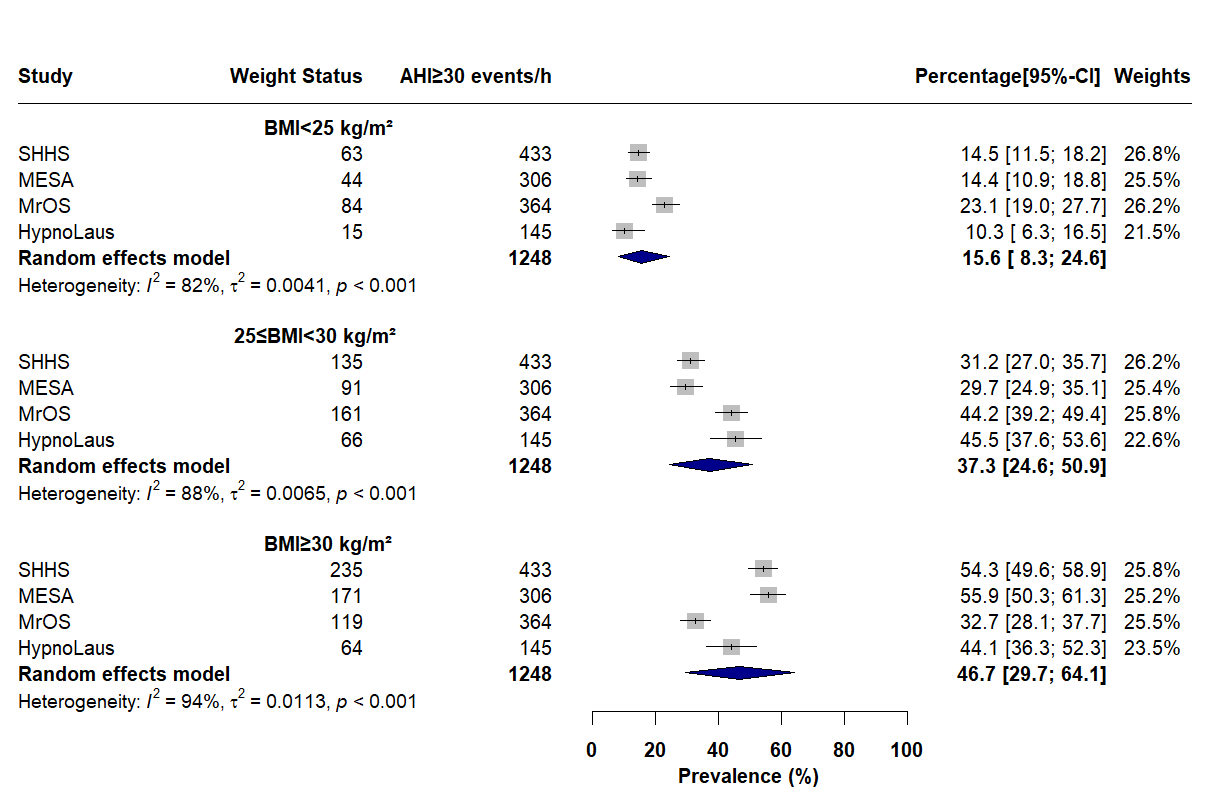


**eFigure 2. Meta prevalence of different weight groups in mild OSA, defined as 5≤AHI<15 events/h. OSA: Obstructive sleep apnea. AHI: apnea-hypopnea index. BMI: body mass index (kg/m^2^). SHHS: Sleep Heart Health Study. MrOS: Osteoporotic Fractures in Men Study. MESA: Multi-Ethnic Study of Atherosclerosis**


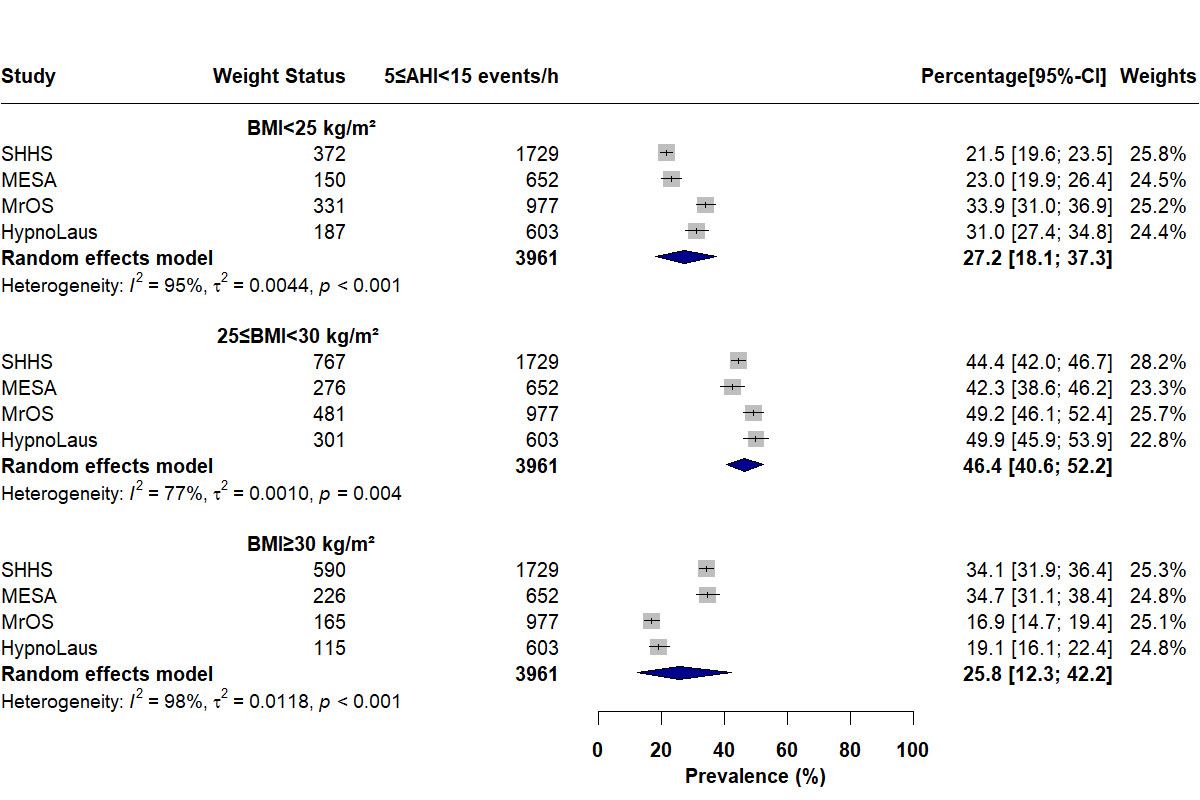


**eFigure 3. Meta prevalence of different weight groups in moderate OSA, defined as 15≤AHI<30 events/h. OSA: Obstructive sleep apnea. AHI: apnea-hypopnea index. BMI: body mass index (kg/m^2^). SHHS: Sleep Heart Health Study. MrOS: Osteoporotic Fractures in Men Study. MESA: Multi-Ethnic Study of Atherosclerosis**


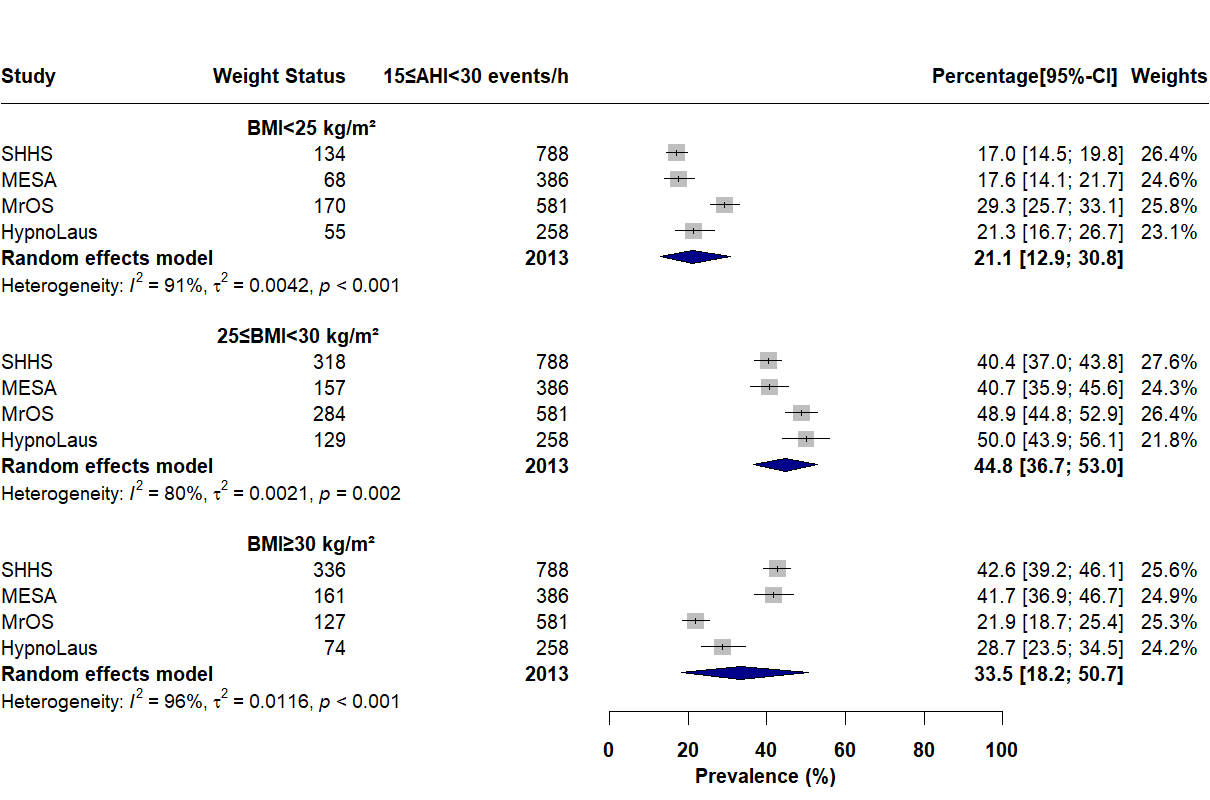


**eFigure 4. Sensitivity analyses by leave-one-out method to investigate the effect of excluding each study on meta prevalence of different weight status defined by BMI in OSA, defined as AHI≥5 events/h, and moderate to severe OSA, defined as AHI≥15 events/h. OSA: Obstructive sleep apnea. AHI: apnea-hypopnea index. BMI: body mass index. SHHS: Sleep Heart Health Study. MrOS: Osteoporotic Fractures in Men Study. MESA: Multi-Ethnic Study of Atherosclerosis.**


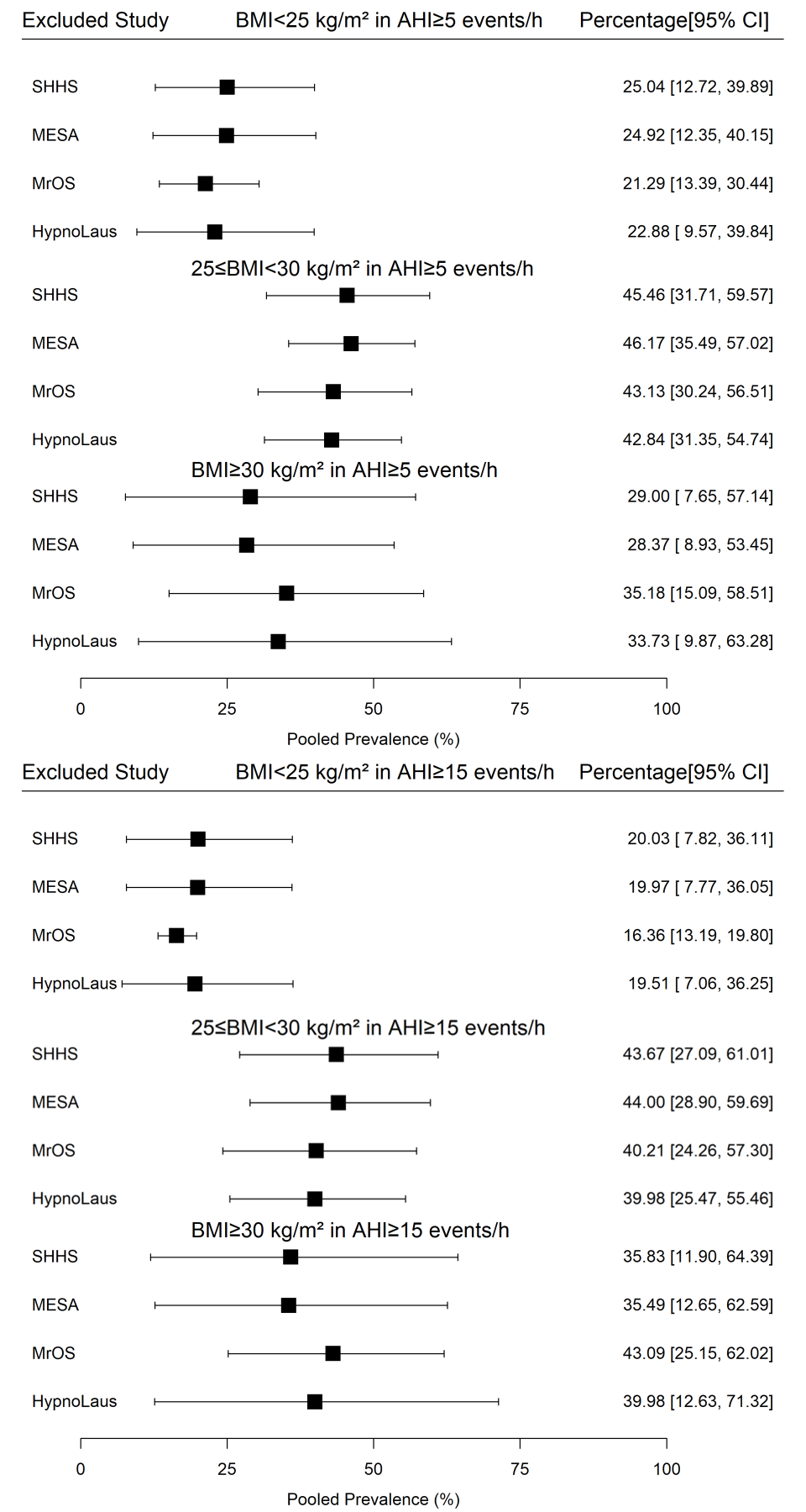

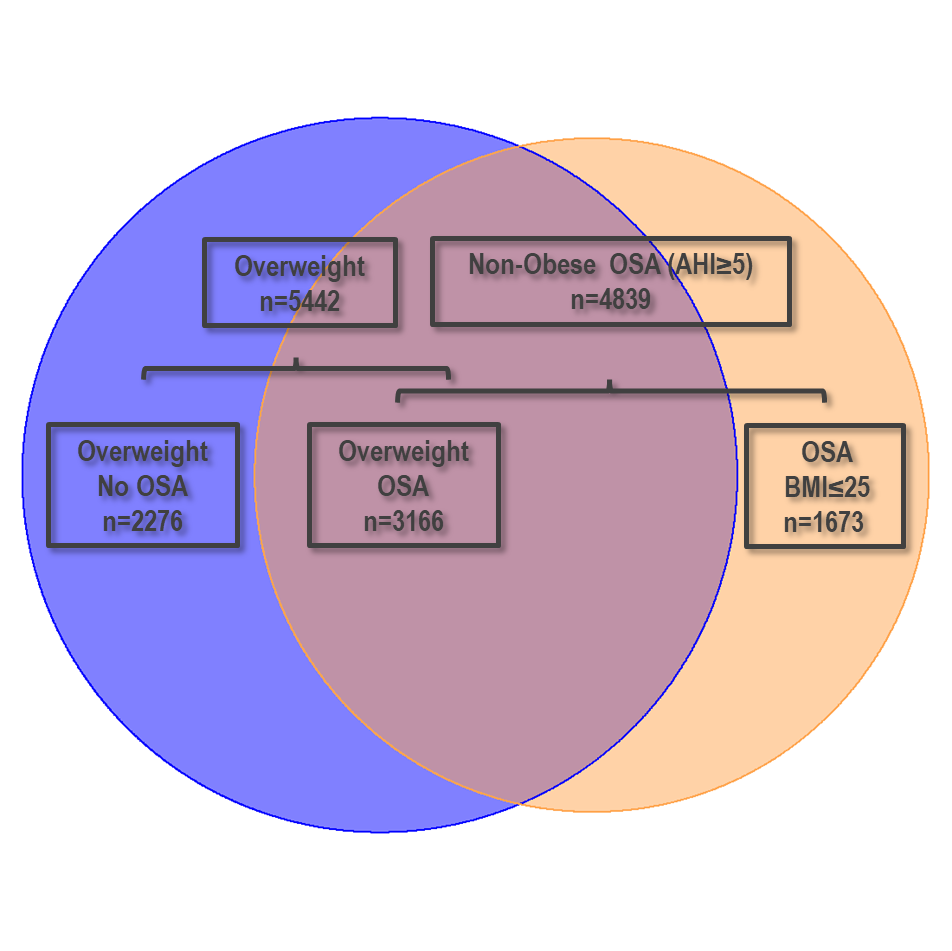


**eFigure 5. Venn diagram to display intersection of OSA** **without obesity defined by AHI≥5 events/h & BMI<30 kg/m^2^ and overweight, defined by 25≤BMI<30 kg/m^2^.**


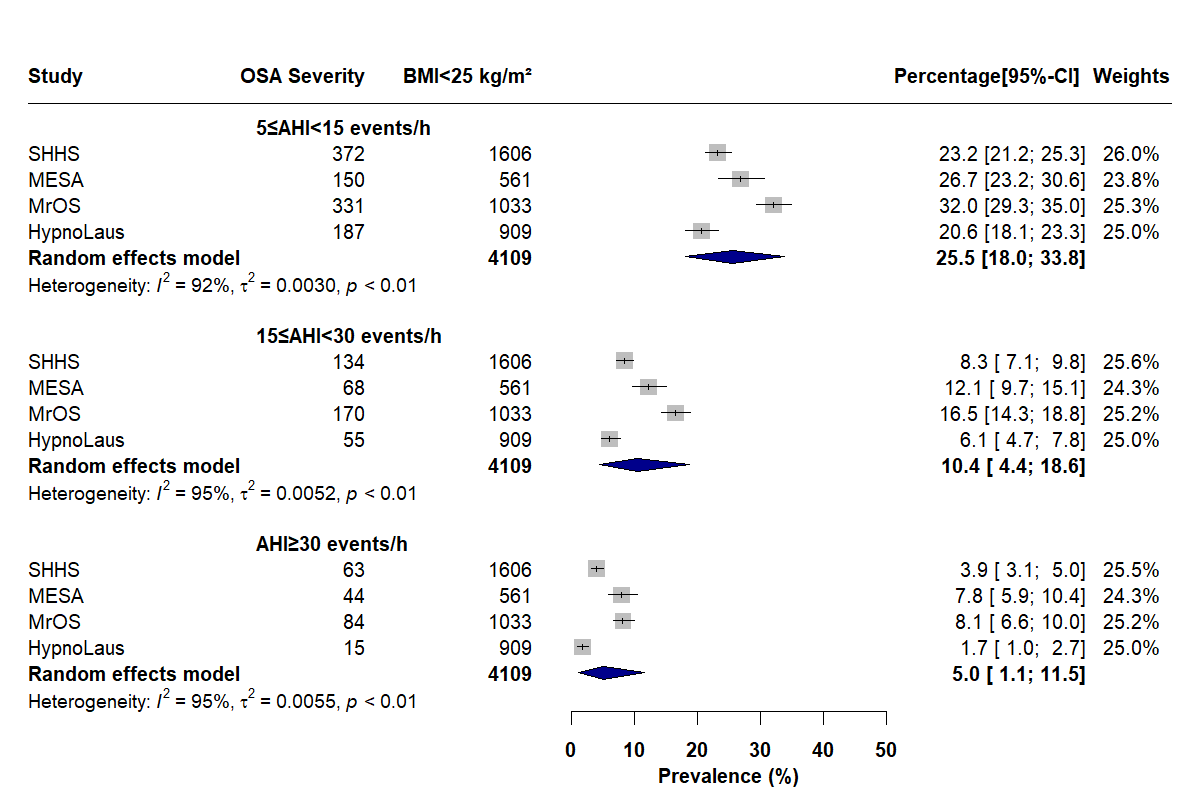


**eFigure 6. Meta prevalence of different OSA categories****, in the group of individuals with underweight/normal weight, defined as BMI<25 kg/m^2^. OSA: Obstructive sleep apnea. AHI: apnea-hypopnea index. BMI: body mass index (kg/m^2^). SHHS: Sleep Heart Health Study. MrOS: Osteoporotic Fractures in Men Study. MESA: Multi-Ethnic Study of Atherosclerosis.**

**eFigure 7. Meta prevalence of different OSA categories in the** **group of individuals with overweight, defined as 25≤BMI<30 kg/m^2^. OSA: Obstructive sleep apnea. AHI: apnea-hypopnea index. BMI: body mass index. SHHS: Sleep Heart Health Study. MrOS: Osteoporotic Fractures in Men Study. MESA: Multi-Ethnic Study of Atherosclerosis.**


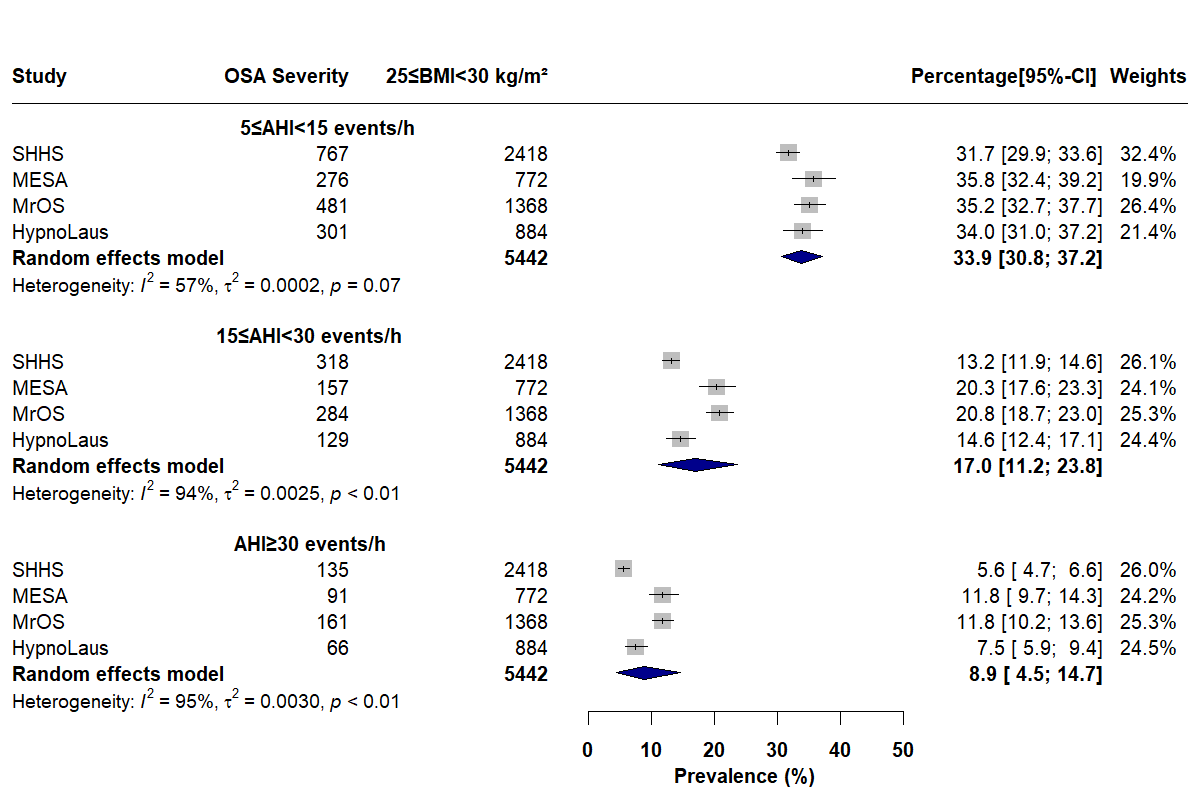


**eFigure 8. Meta prevalence of different OSA categories in the group** **of individuals with obesity, defined as BMI≥30 kg/m^2^.OSA: Obstructive sleep apnea. AHI: apnea-hypopnea index. BMI: body mass index. SHHS: Sleep Heart Health Study. MrOS: Osteoporotic Fractures in Men Study. MESA: Multi-Ethnic Study of Atherosclerosis**


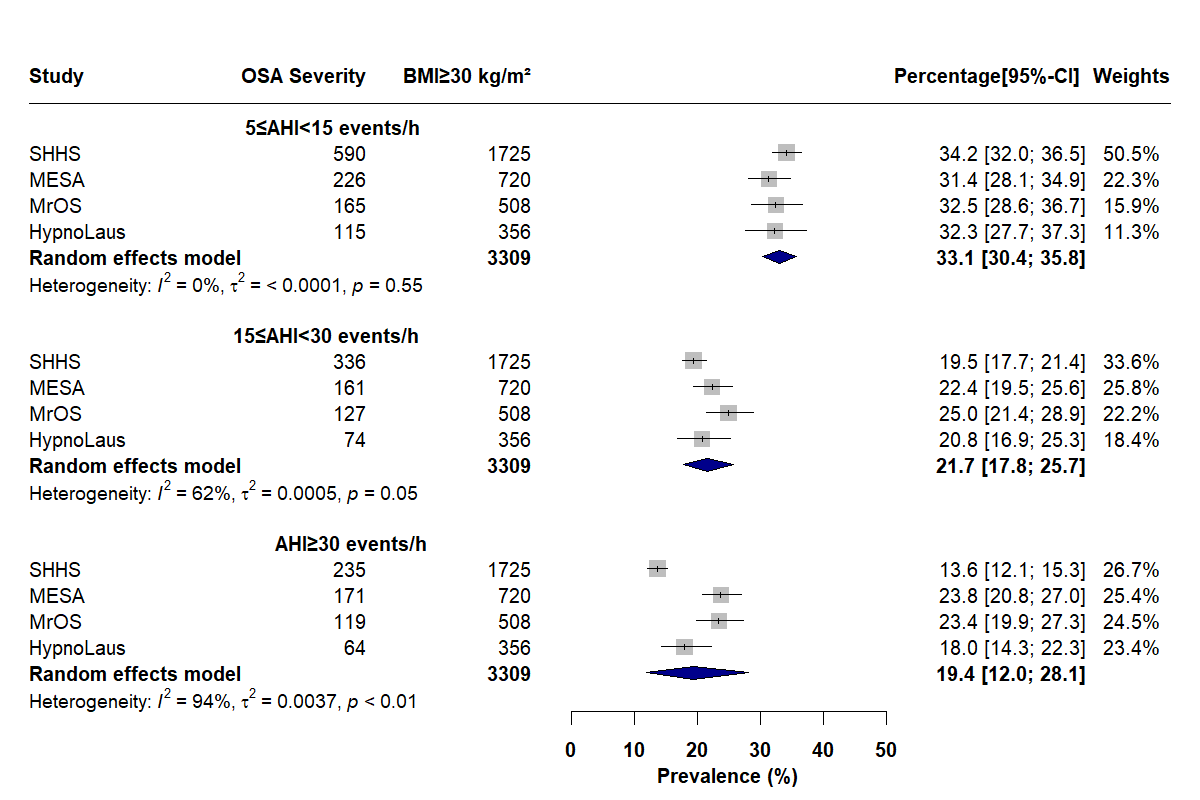


**eFigure 9. Sensitivity analyses by leave-one-out method to investigate the effect of excluding each study on meta prevalence of OSA, defined as AHI≥5 events/h, and moderate to severe OSA, defined as AHI≥15 events/h, in** **the group of individuals with obesity, defined by BMI≥30 kg/m^2^ (two first forest plots) or with overweight; defined by 25≥BMI>30 kg/m^2^ (two last forest plots). OSA: Obstructive sleep apnea. AHI: apnea-hypopnea index. BMI: body mass index. SHHS: Sleep Heart Health Study. MrOS: Osteoporotic Fractures in Men Study. MESA: Multi-Ethnic Study of Atherosclerosis.**


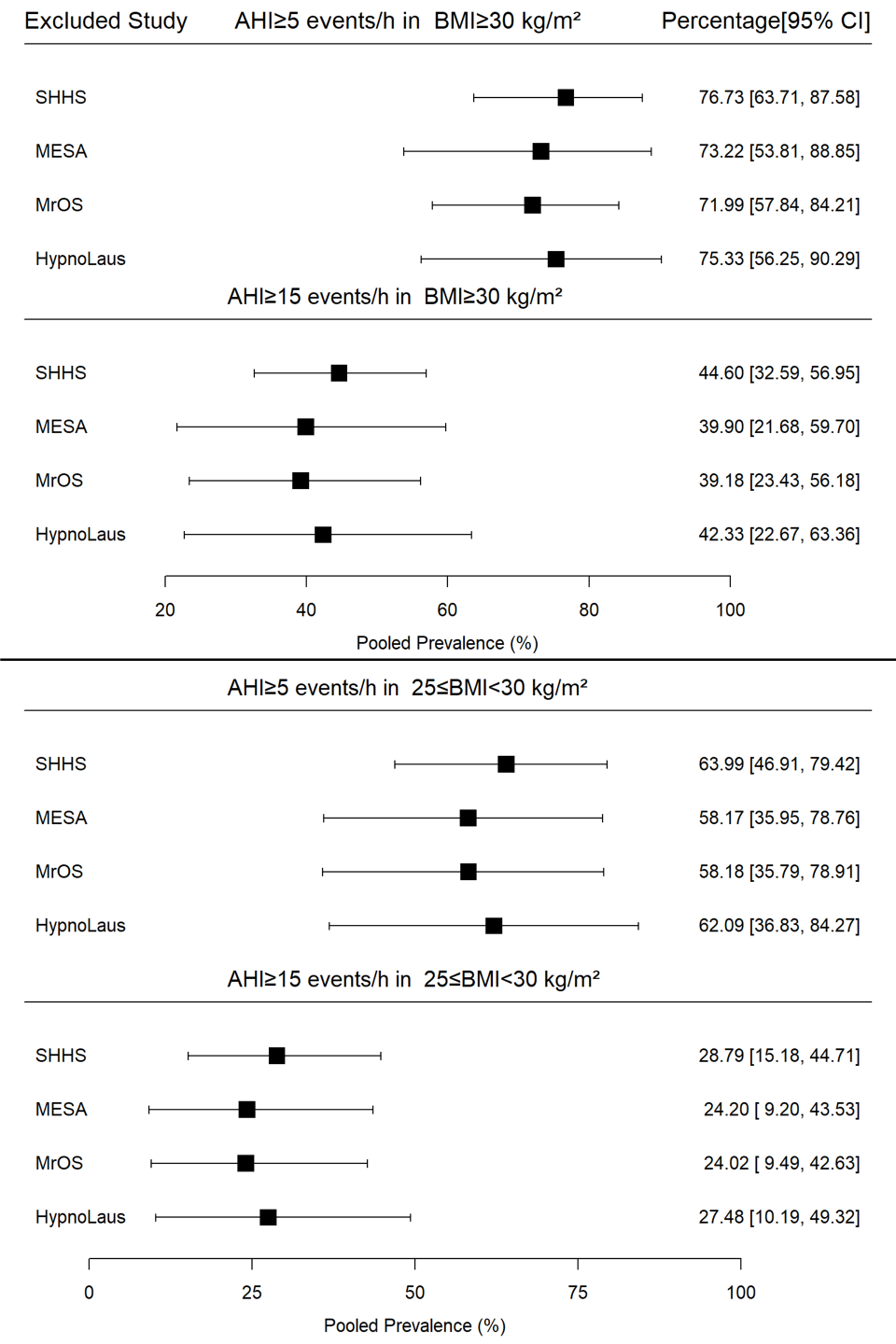


**eFigure 10. Sex vs age subgroup Meta prevalence of obesity, defined as BMI≥30 kg/m^2^, in OSA, defined as AHI≥5 events/h. OSA: Obstructive sleep apnea. AHI: apnea-hypopnea index. BMI: body mass index. SHHS: Sleep Heart Health Study. MrOS: Osteoporotic Fractures in Men Study. MESA: Multi-Ethnic Study of Atherosclerosis.**


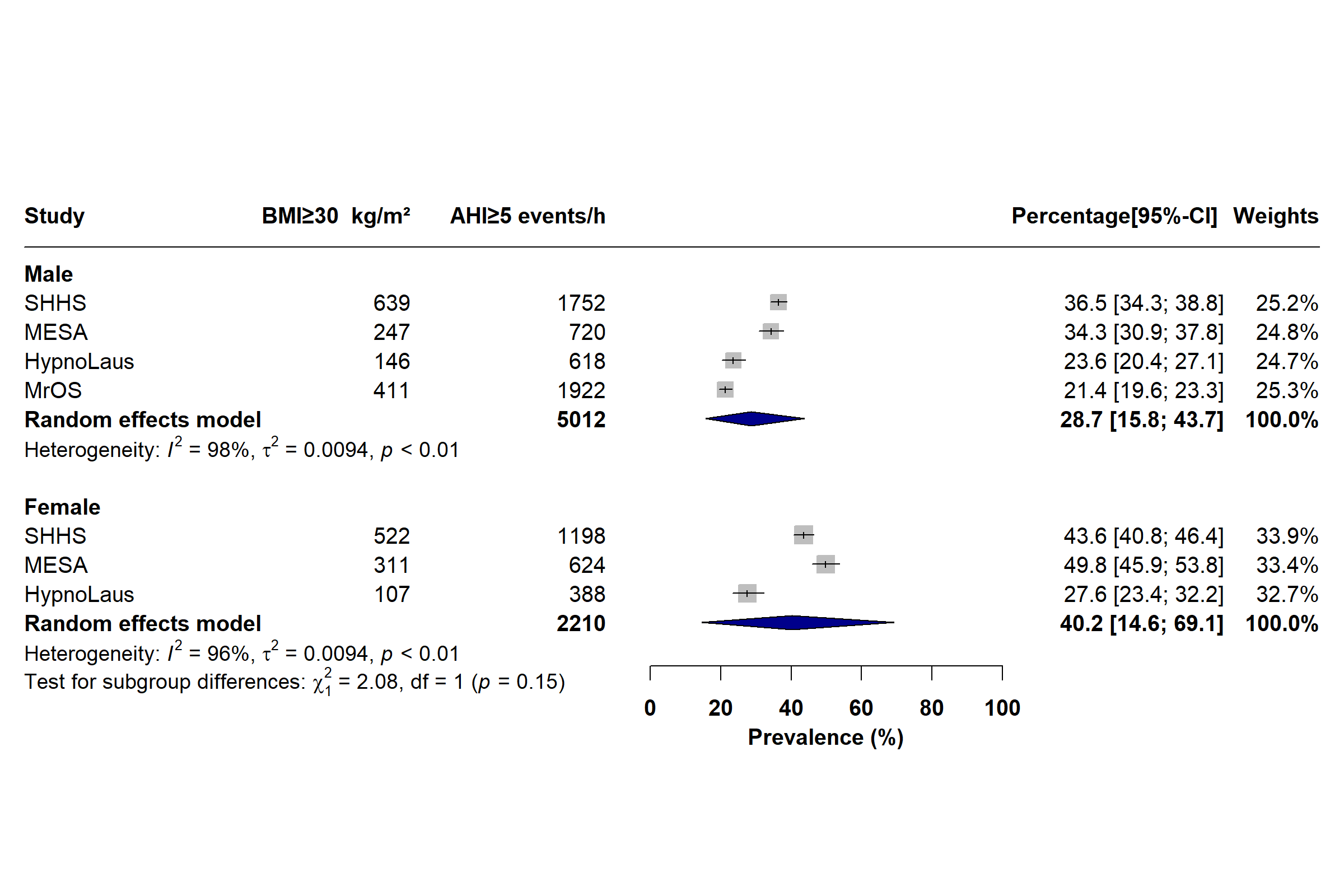

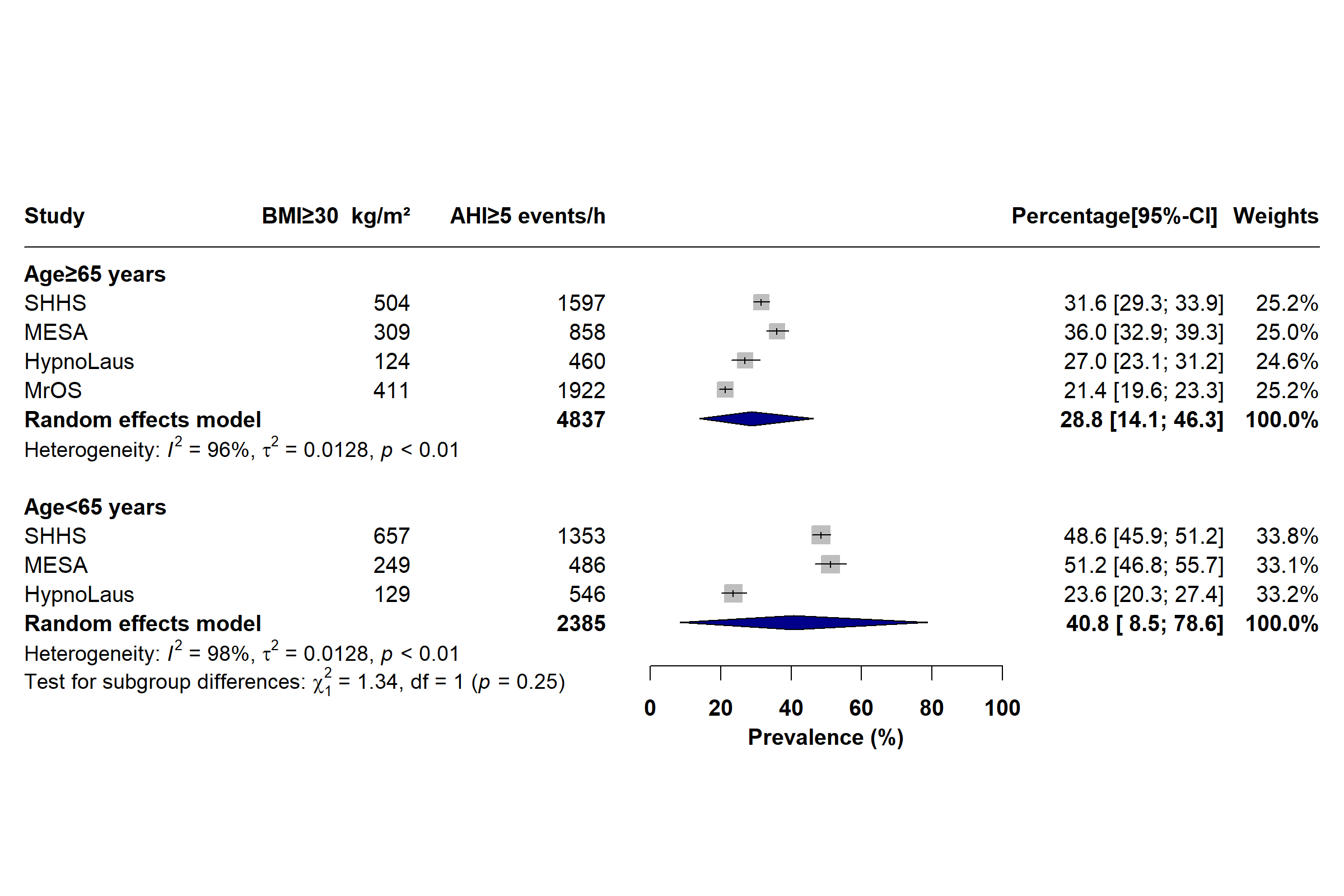


**Age < 65 years Age ≥ 65 years**

**Female Male**

**eFigure 11. Pie charts to indicate the prevalence of weight groups in sex or age subgroups of OSA defined by 5≤AHI<15 events/h, 15≤AHI<30 events/h and AHI≥30 events/h. Weight groups are shown in light color (BMI<25 kg/m^2^), moderate color (25≤BMI<30 kg/m^2^) and dark color (BMI≥30 kg/m^2^) in each OSA subgroup of SHHS. OSA: Obstructive sleep apnea. AHI: apnea-hypopnea index. BMI: body mass index (kg/m^2^). N: number. SHHS: Sleep Heart Health Study.**


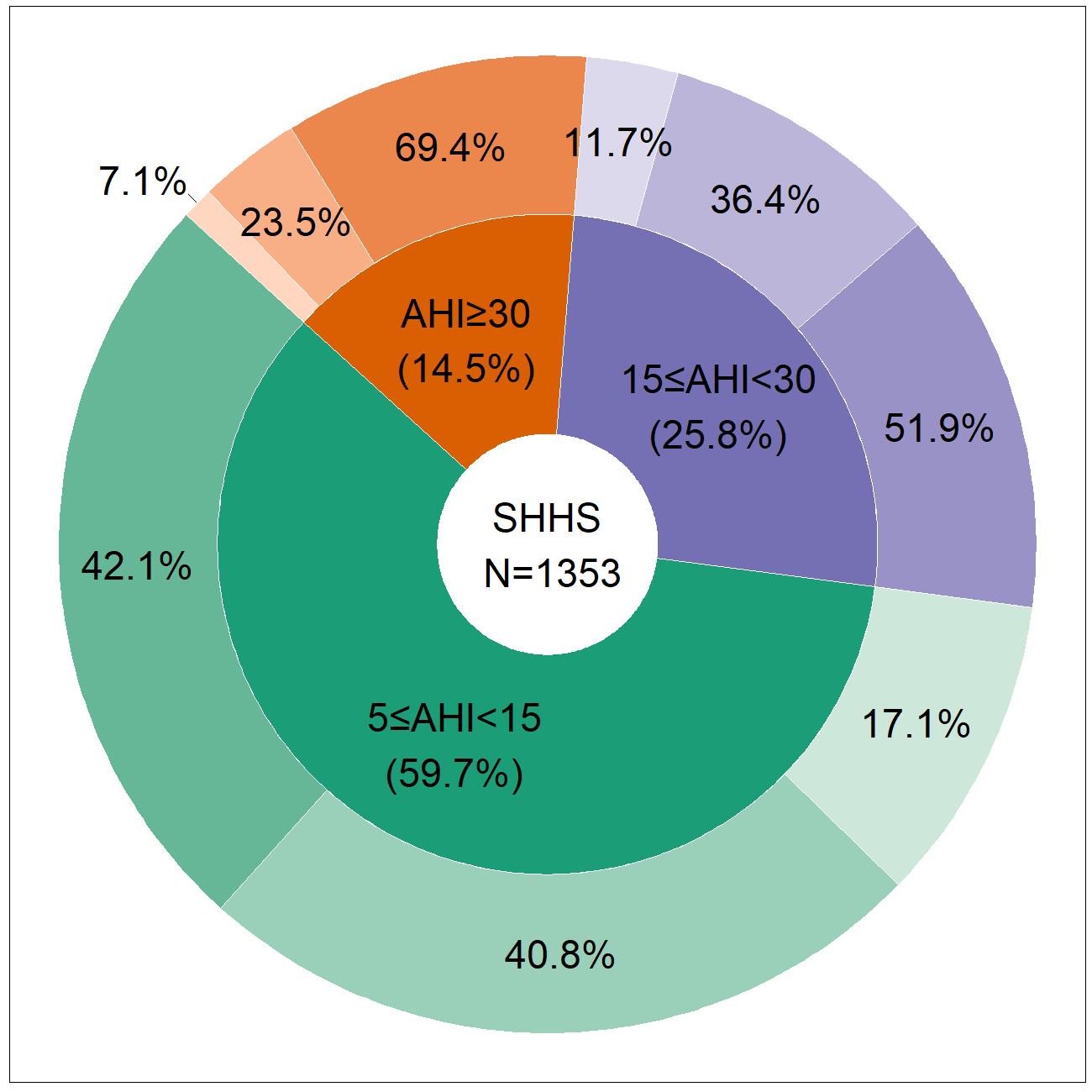

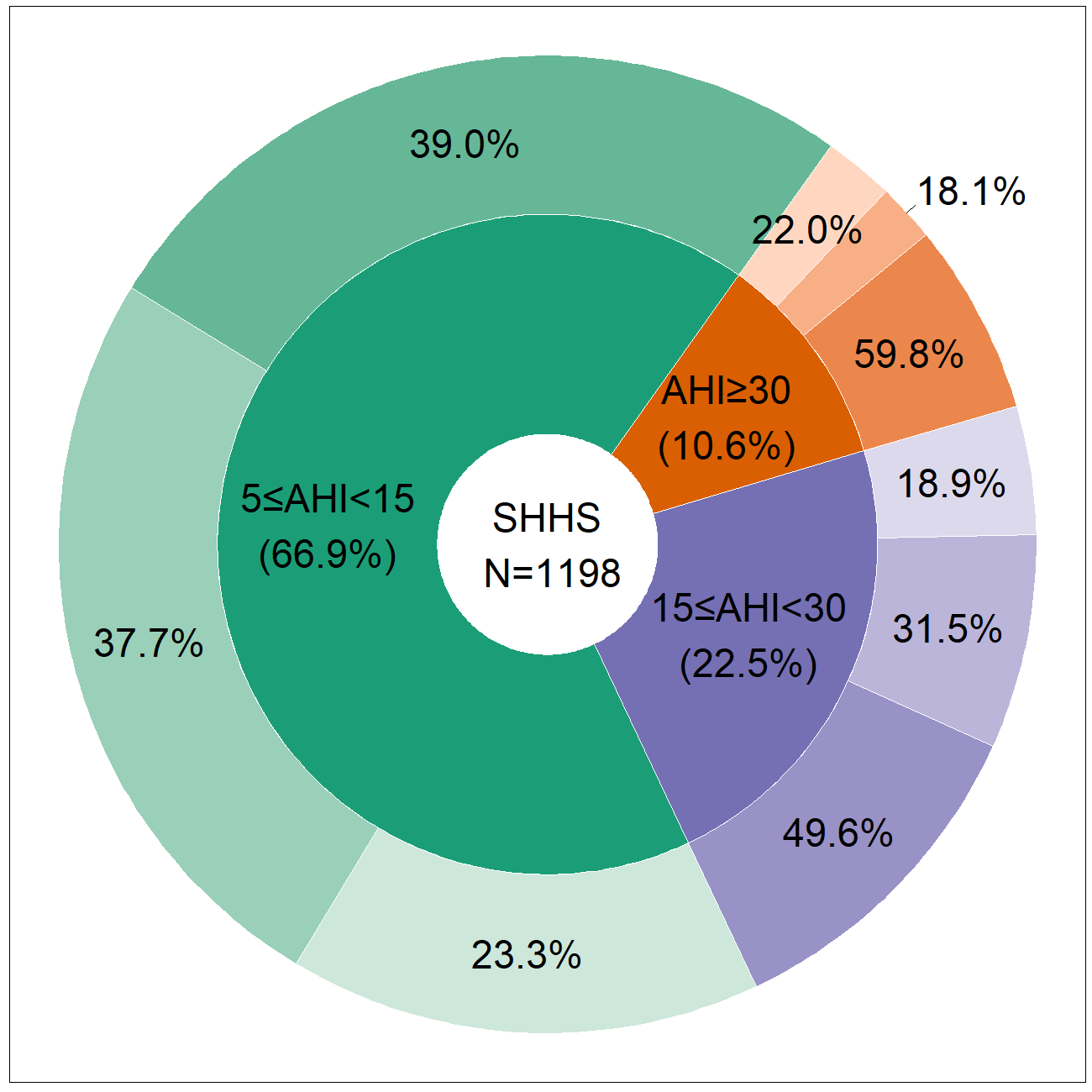

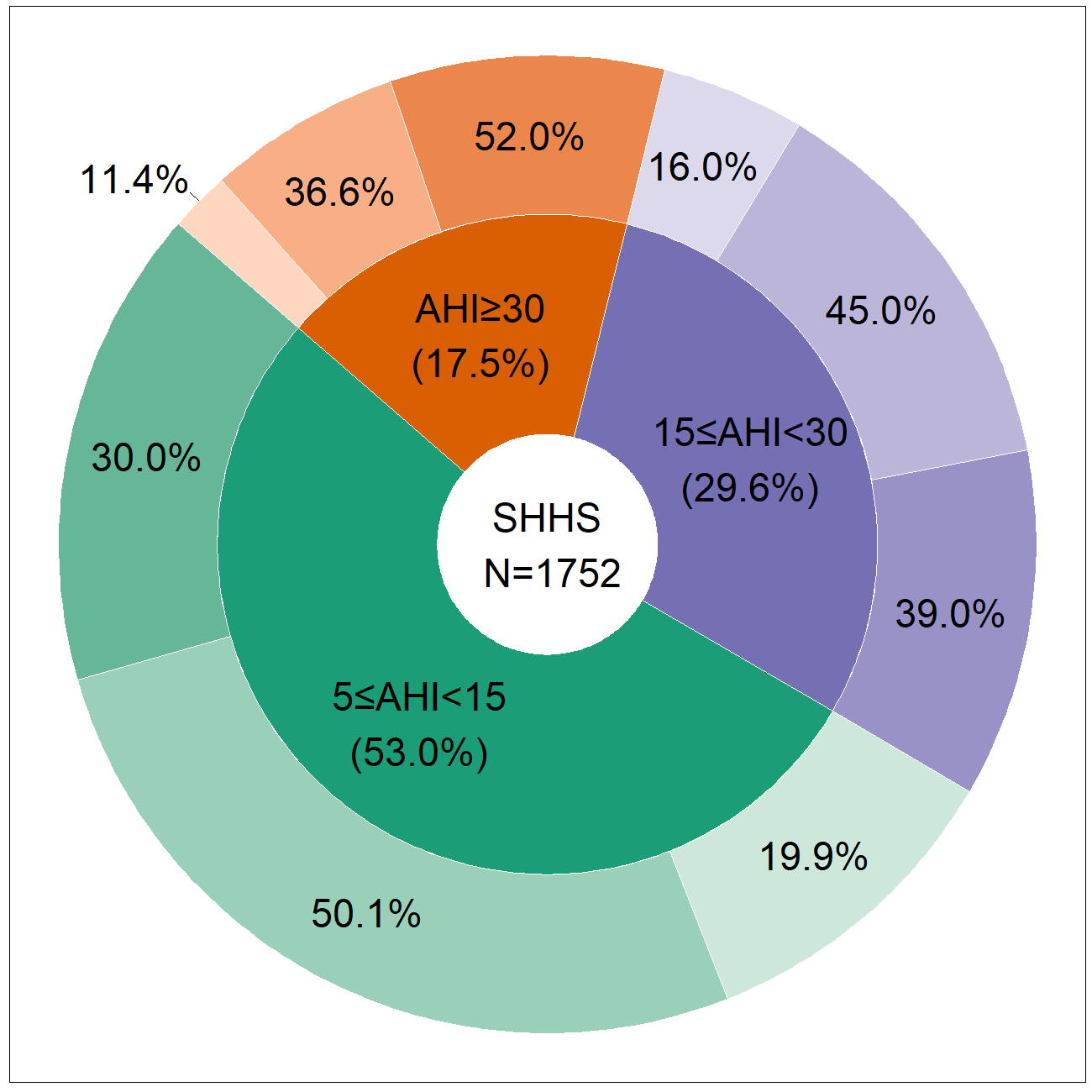

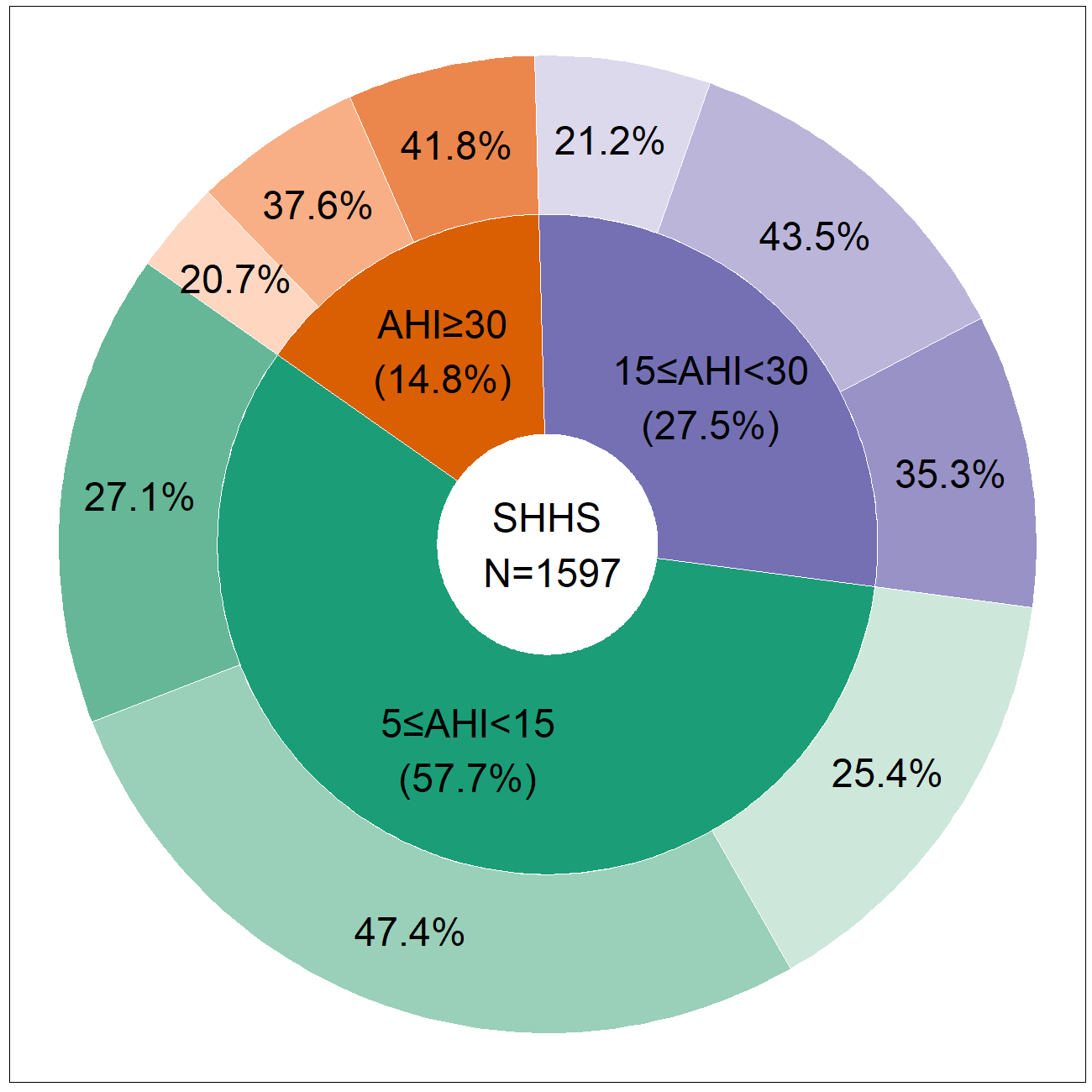


**Female Male**

**eFigure 12. Pie charts to indicate the prevalence of weight groups in sex or age subgroups of OSA defined by 5≤AHI<15 events/h, 15≤AHI<30 events/h and AHI≥30 events/h. Weight groups are shown in light color (BMI<25 kg/m^2^), moderate color (25≤BMI<30 kg/m^2^) and dark color (BMI≥30 kg/m^2^) in each OSA subgroup of MESA. OSA: Obstructive sleep apnea. AHI: apnea-hypopnea index. BMI: body mass index (kg/m^2^). N: number. MESA: Multi-Ethnic Study of Atherosclerosis.**

**Age < 65 years Age ≥ 65 years**


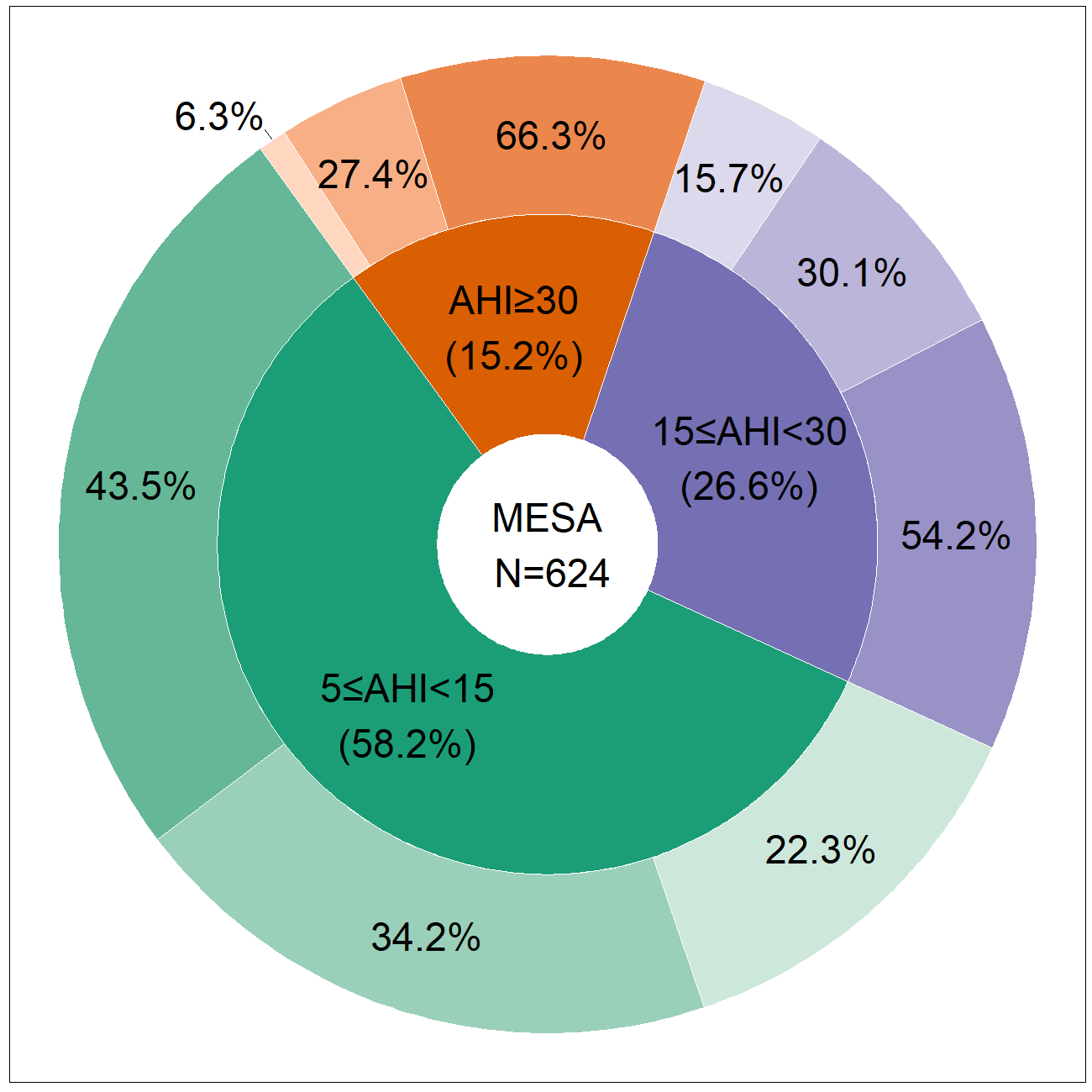

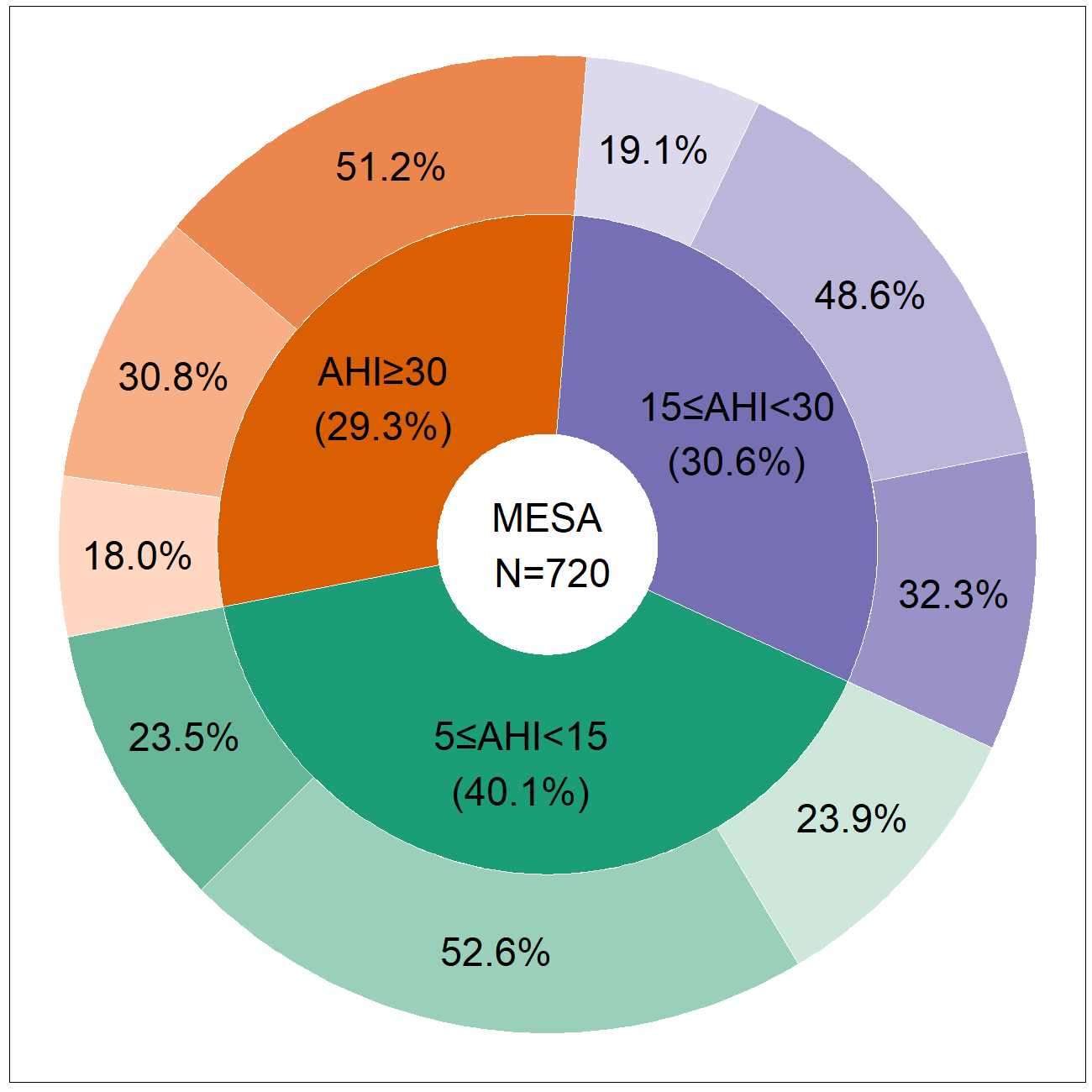

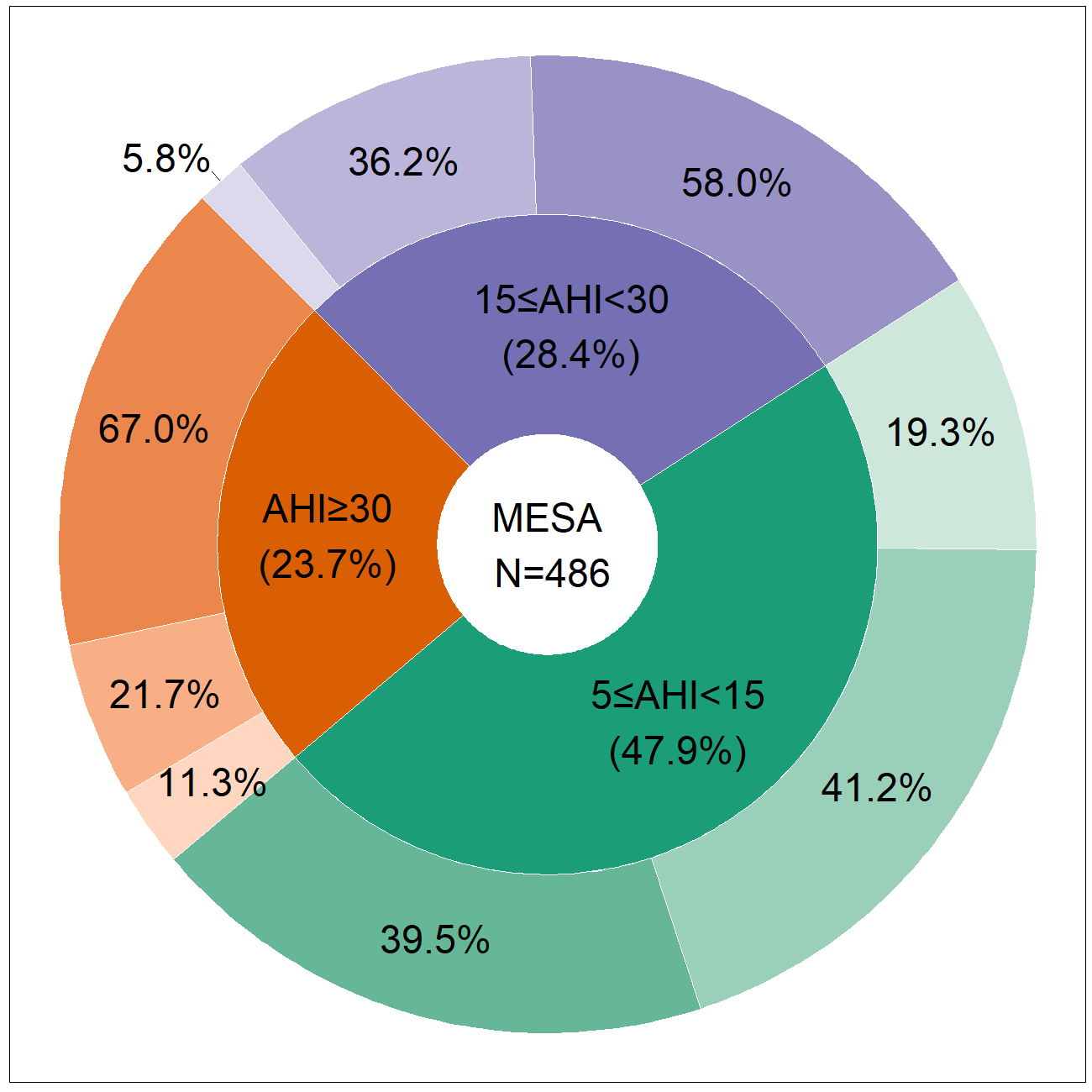

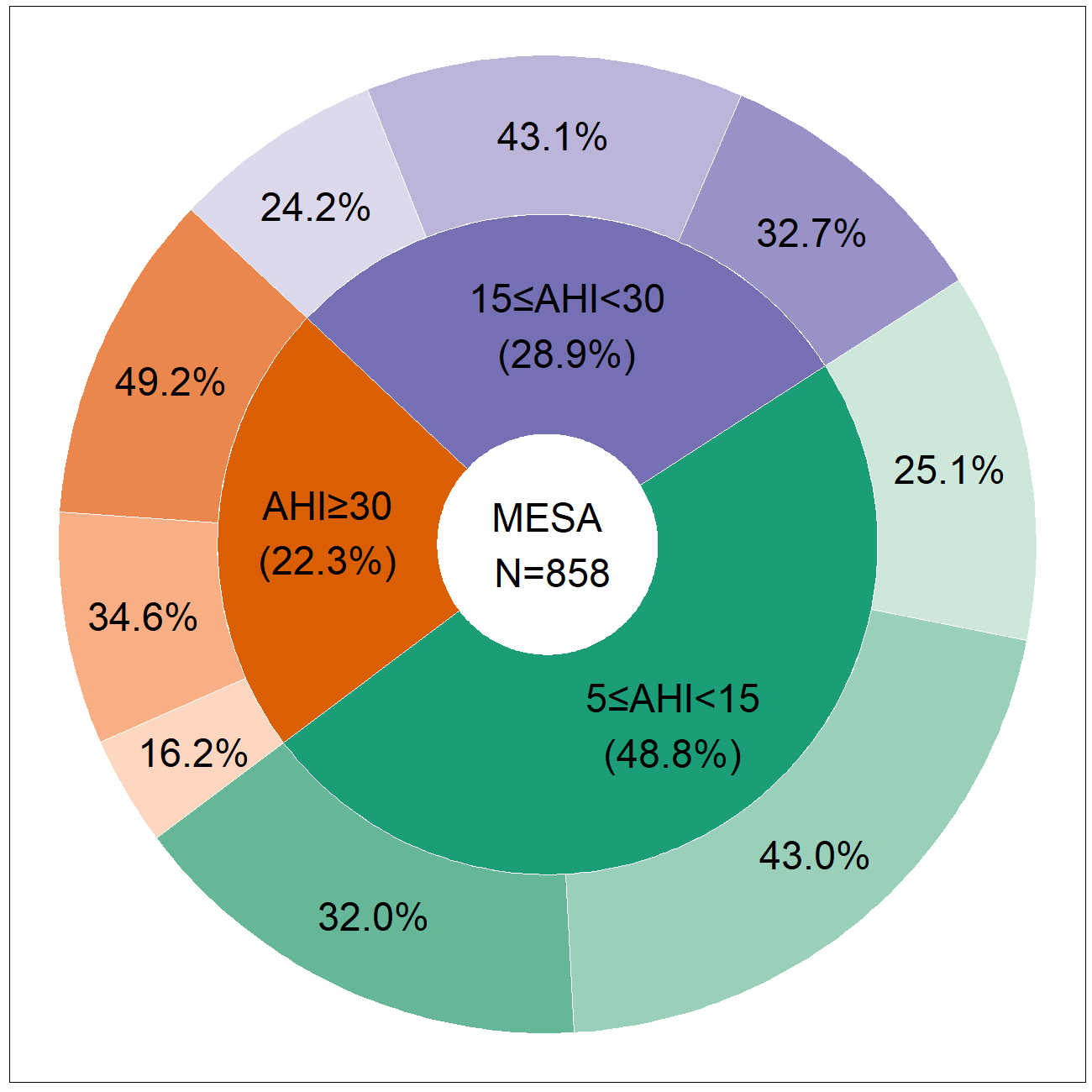

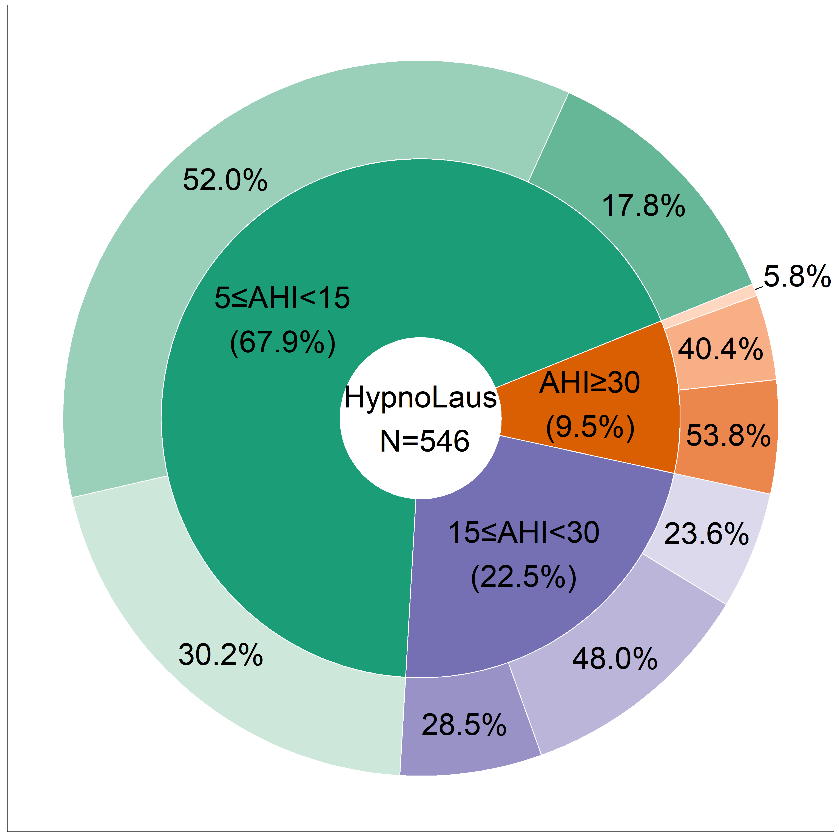

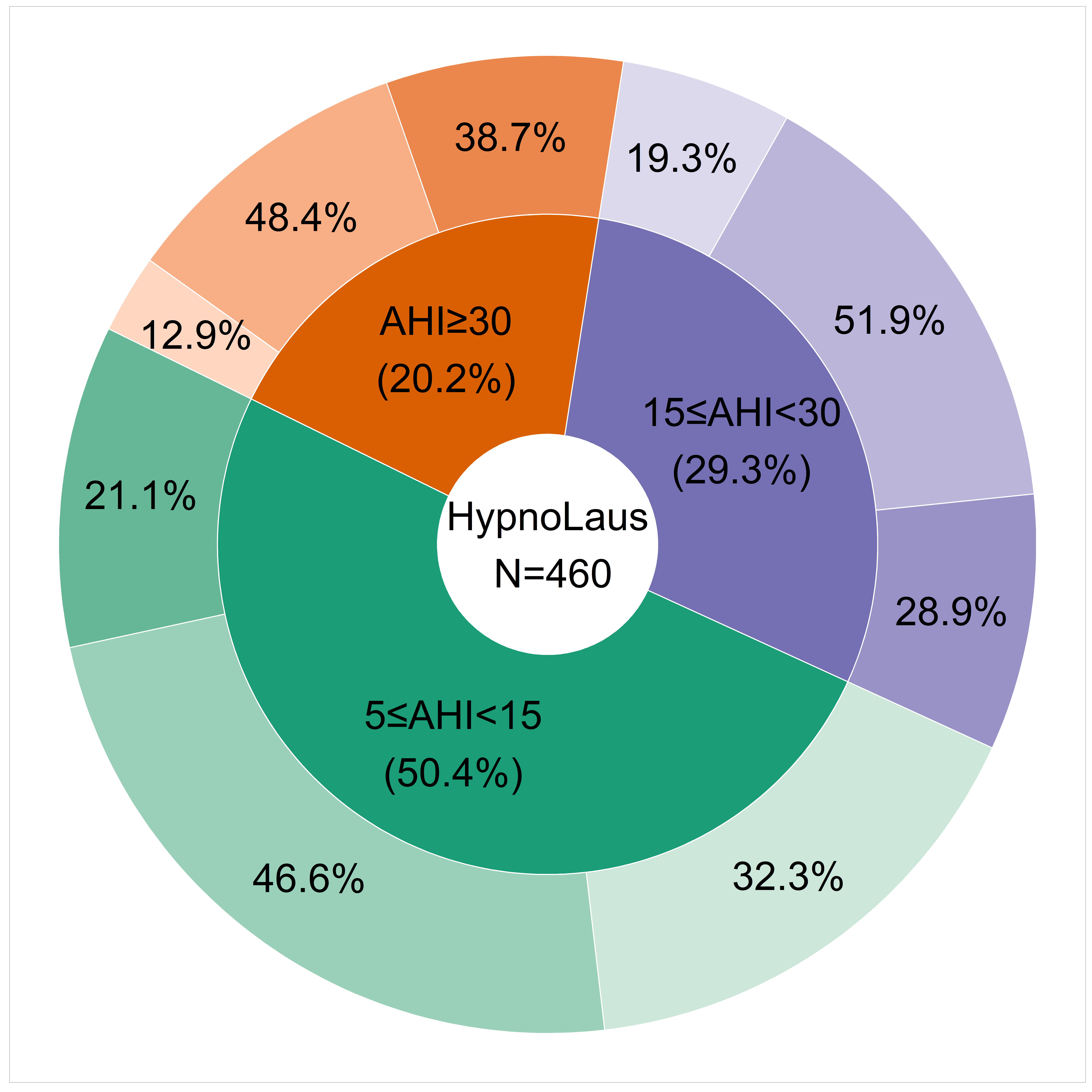


**Female Male**

**eFigure 13. Pie charts to indicate the prevalence of weight groups in sex or age subgroups of OSA defined by 5≤AHI<15 events/h, 15≤AHI<30 events/h and AHI≥30 events/h. Weight groups are shown in light color (BMI<25 kg/m^2^), moderate color (25≤BMI<30 kg/m^2^) and dark color (BMI≥30 kg/m^2^) in each OSA subgroup of HypnoLaus study. OSA: Obstructive sleep apnea. AHI: apnea-hypopnea index. BMI: body mass index (kg/m^2^). N: number.**

**Age < 65 years Age ≥ 65 years**


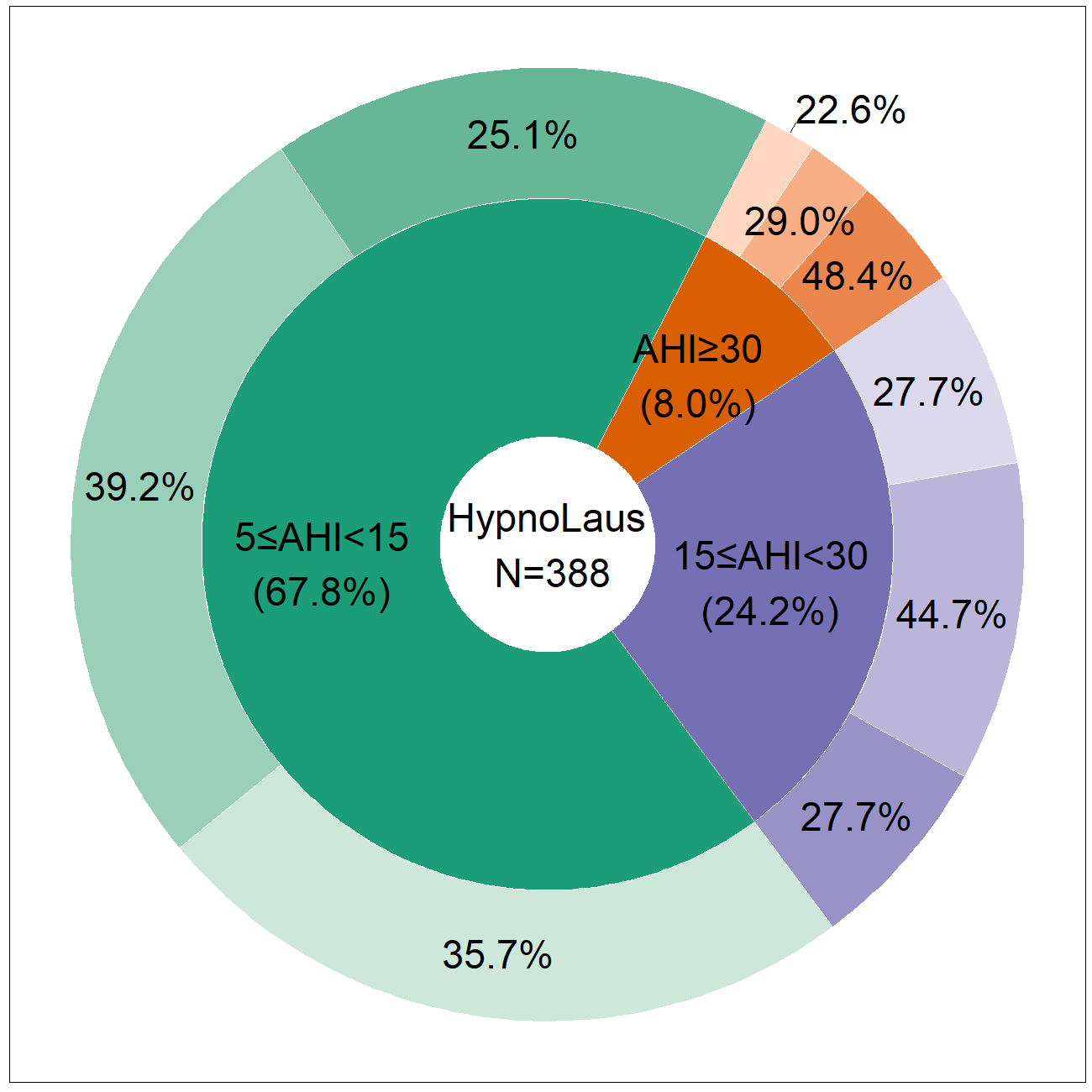

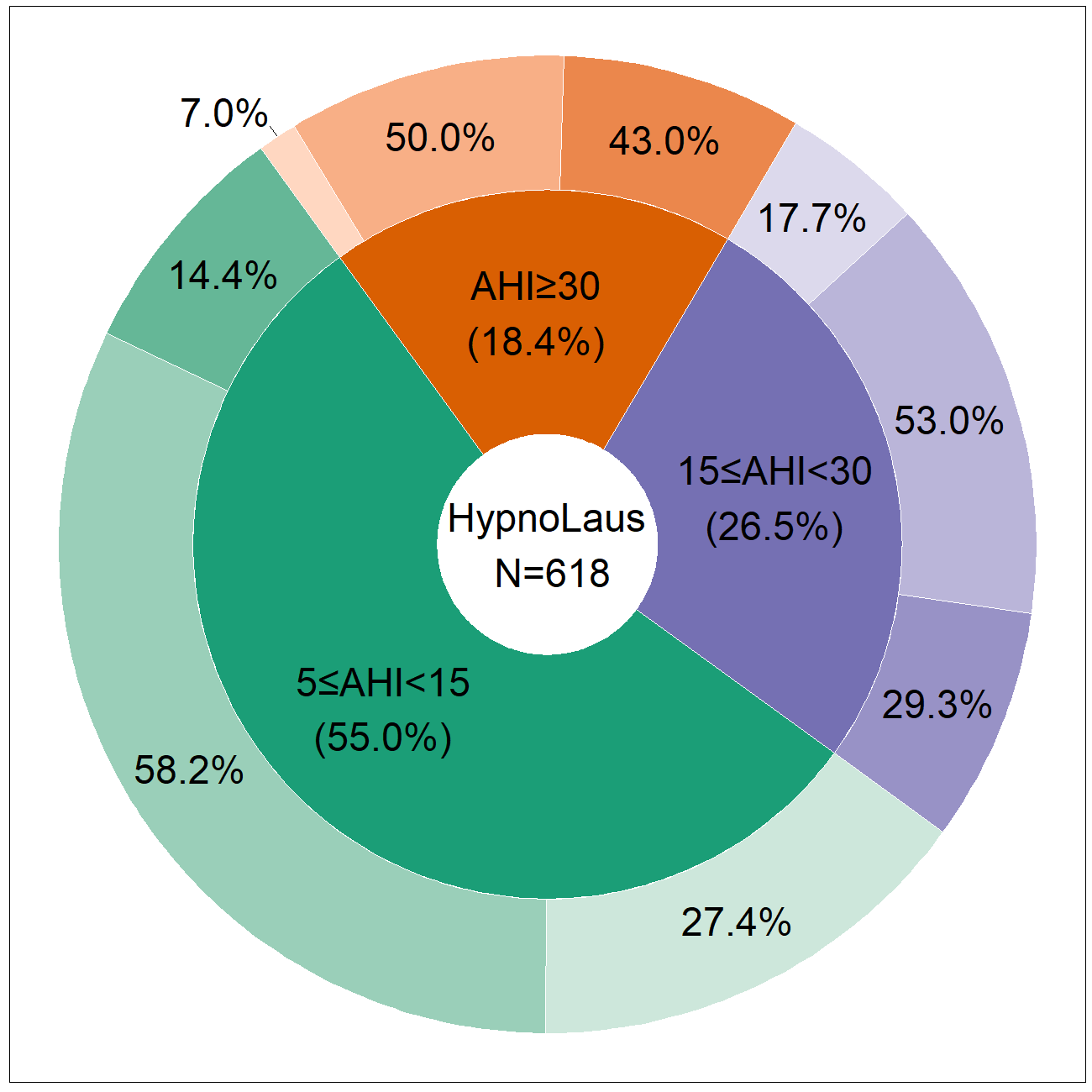

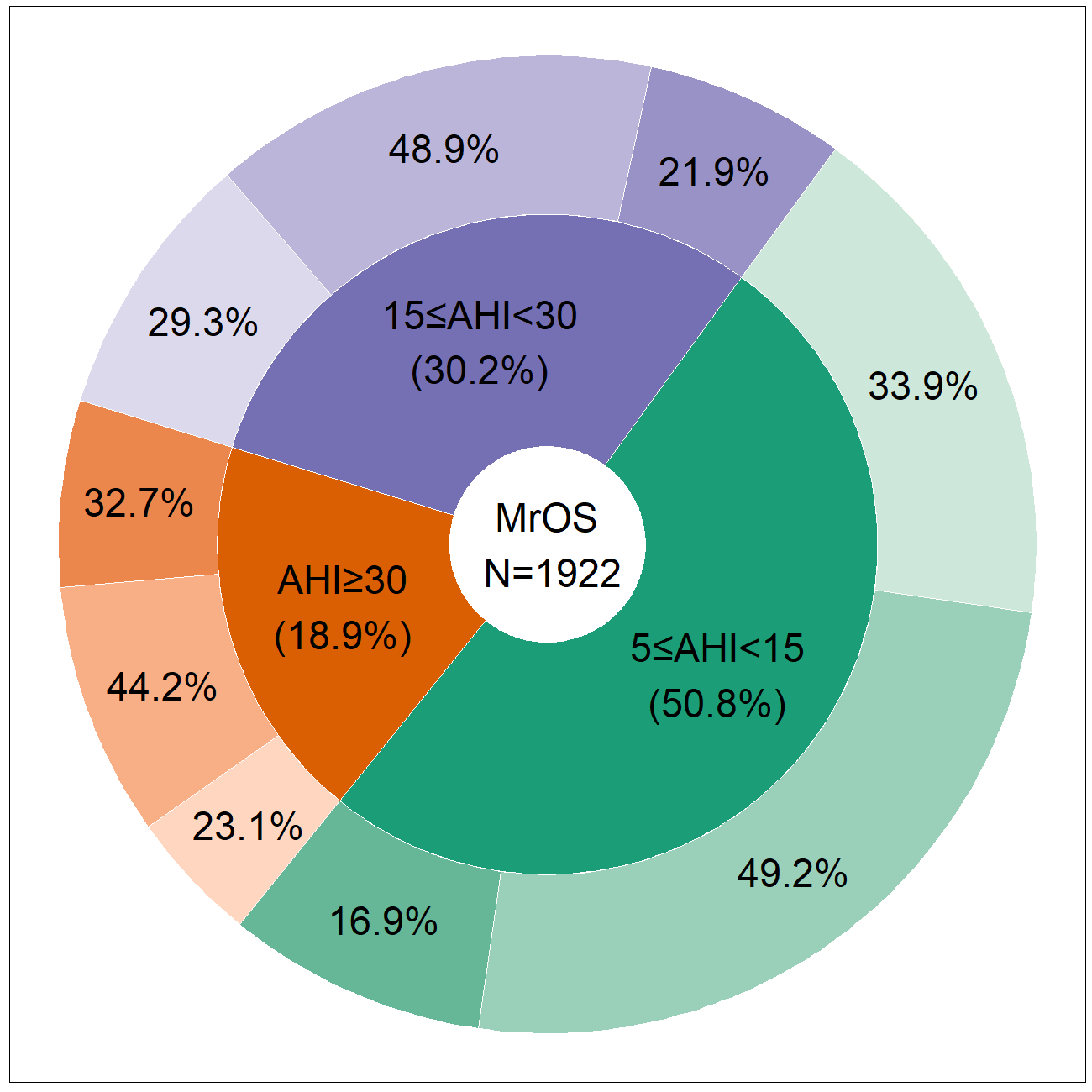

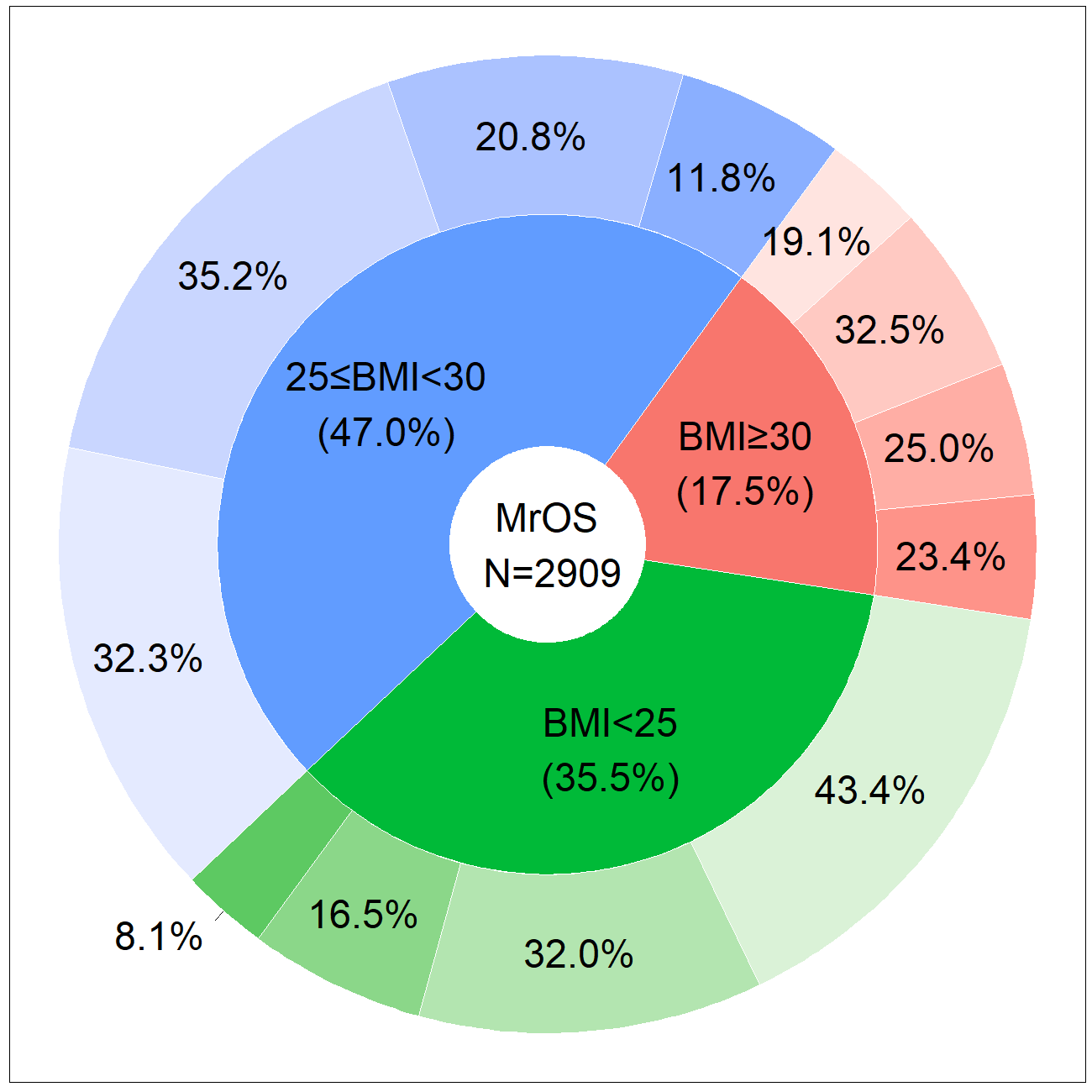


**eFigure 14. A. Pie chart to indicate the prevalence of weight groups in OSA defined by 5≤AHI<15 events/h, 15≤AHI<30 events/h and AHI≥30 events/h. Weight groups are shown in light color (BMI<25 kg/m^2^), moderate color (25≤BMI<30 kg/m^2^) and dark color (BMI≥30 kg/m^2^) in each OSA subgroup of MrOS study. B. Pie chart to indicate the prevalence of different OSA severities in weight groups of MrOS. OSA severities are shown from light to dark by AHI<5 events/h, 5≤AHI<15 events/h, 15≤AHI<30 events/h and AHI≥30 events/h. AHI: apnea-hypopnea index. BMI: body mass index (kg/m^2^). N: number. MrOS: Osteoporotic Fractures in Men Study.**

**A**

**B**

**Female Male**

**eFigure 15. Pie charts to indicate the prevalence of different OSA severities in sex or age subgroups of weight groups in SHHS. OSA severities are shown from light to dark by AHI<5 events/h, 5≤AHI<15 events/h, 15≤AHI<30 events/h and AHI≥30 events/h. AHI: apnea-hypopnea index. BMI: body mass index (kg/m^2^). N: number. SHHS: Sleep Heart Health Study.**

**Age < 65 years Age ≥ 65 years**


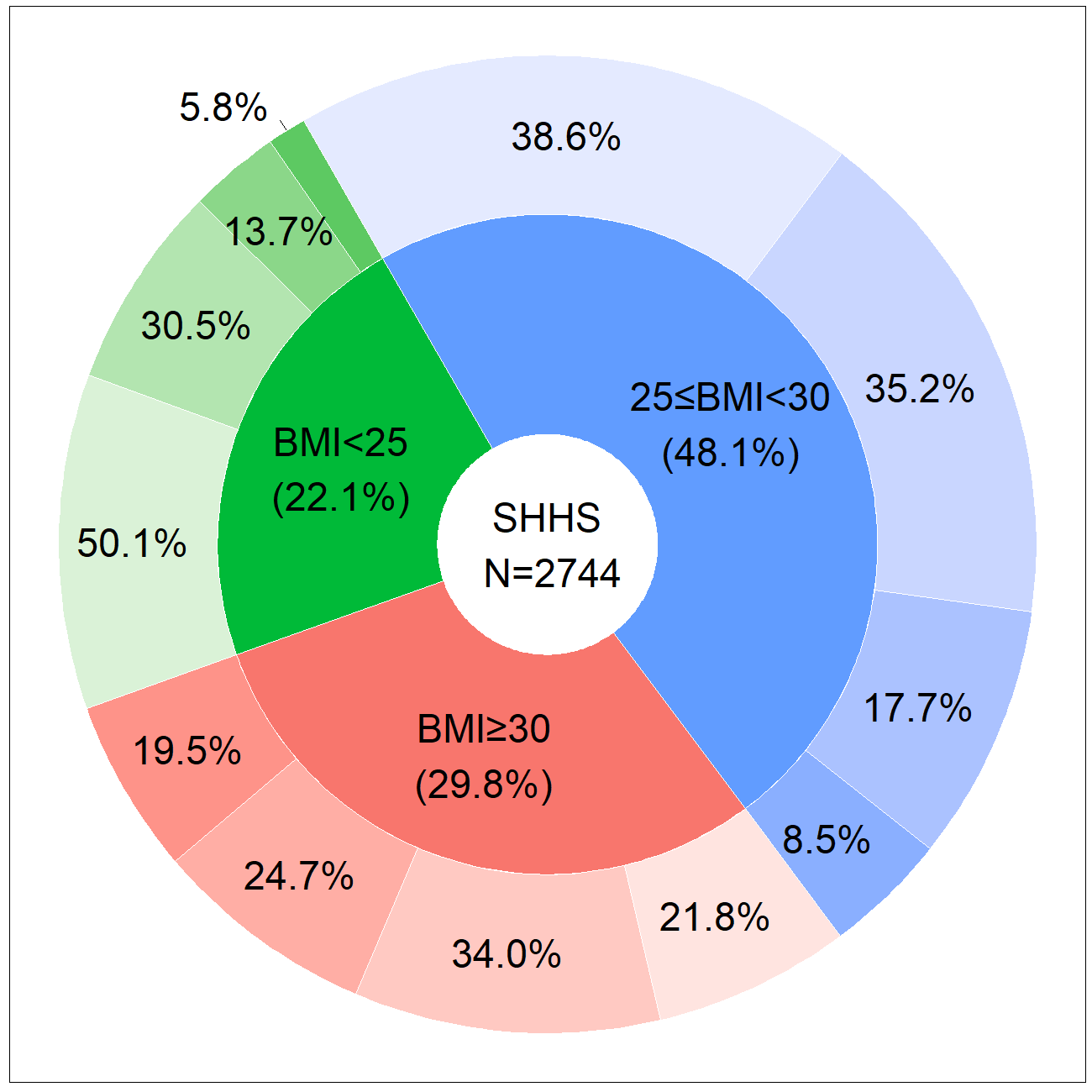

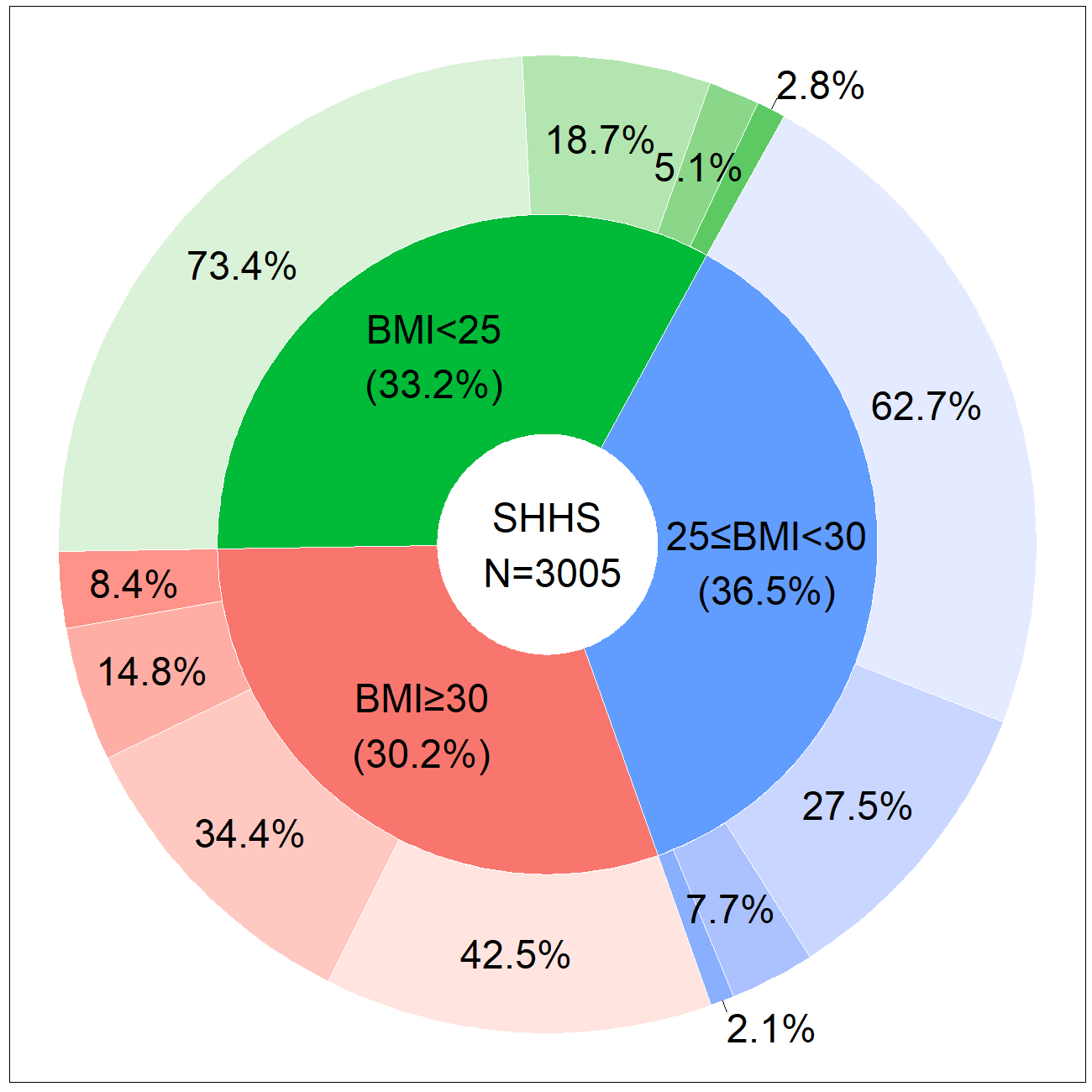

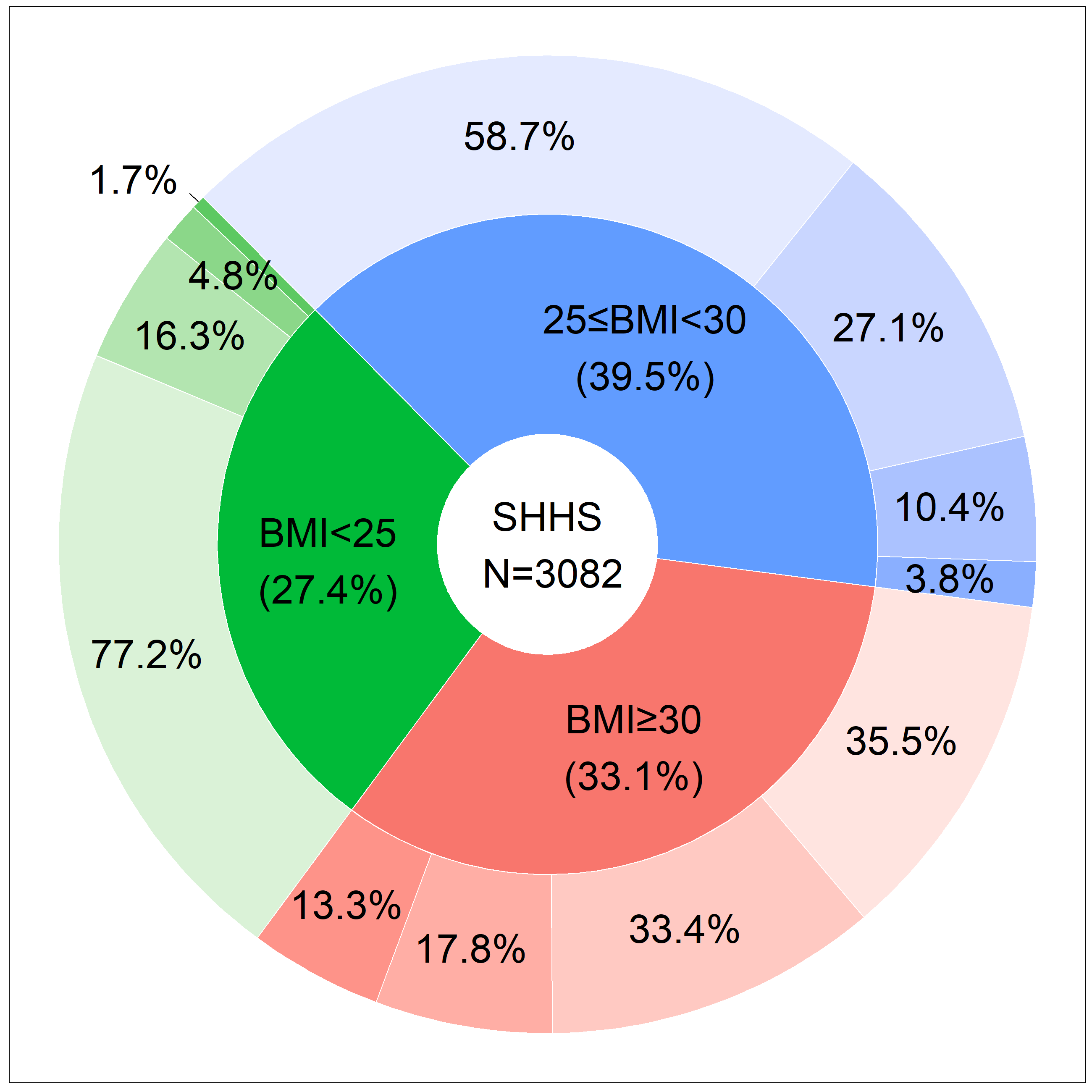

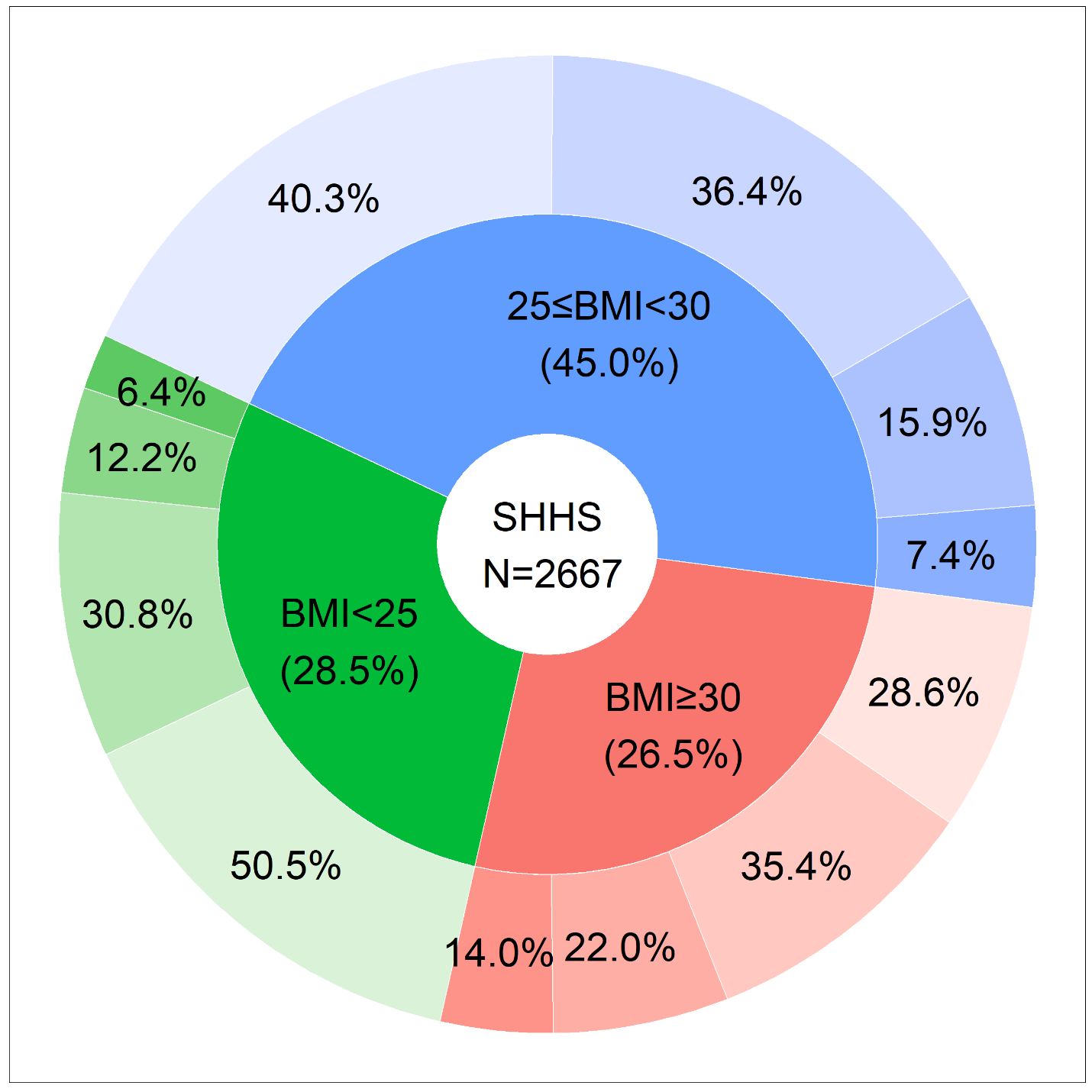


**Female Male**

**eFigure 16. Pie charts to indicate the prevalence of different OSA severities in sex or age subgroups of weight groups in MESA. OSA severities are shown from light to dark by AHI<5 events/h, 5≤AHI<15 events/h, 15≤AHI<30 events/h and AHI≥30 events/h. AHI: apnea-hypopnea index. BMI: body mass index (kg/m^2^). N: number. MESA: Multi-Ethnic Study of Atherosclerosis.**

**Age < 65 years Age ≥ 65 years**


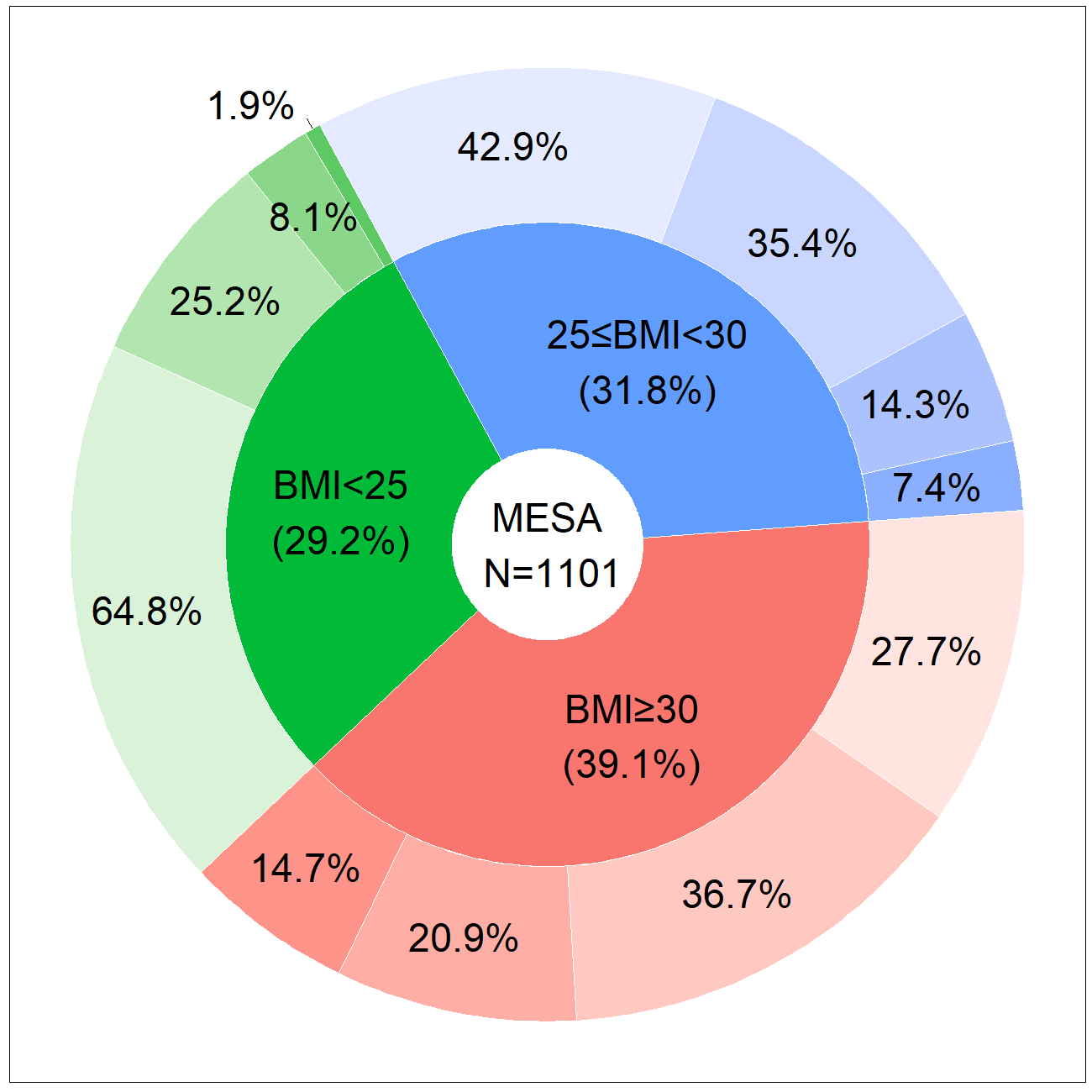

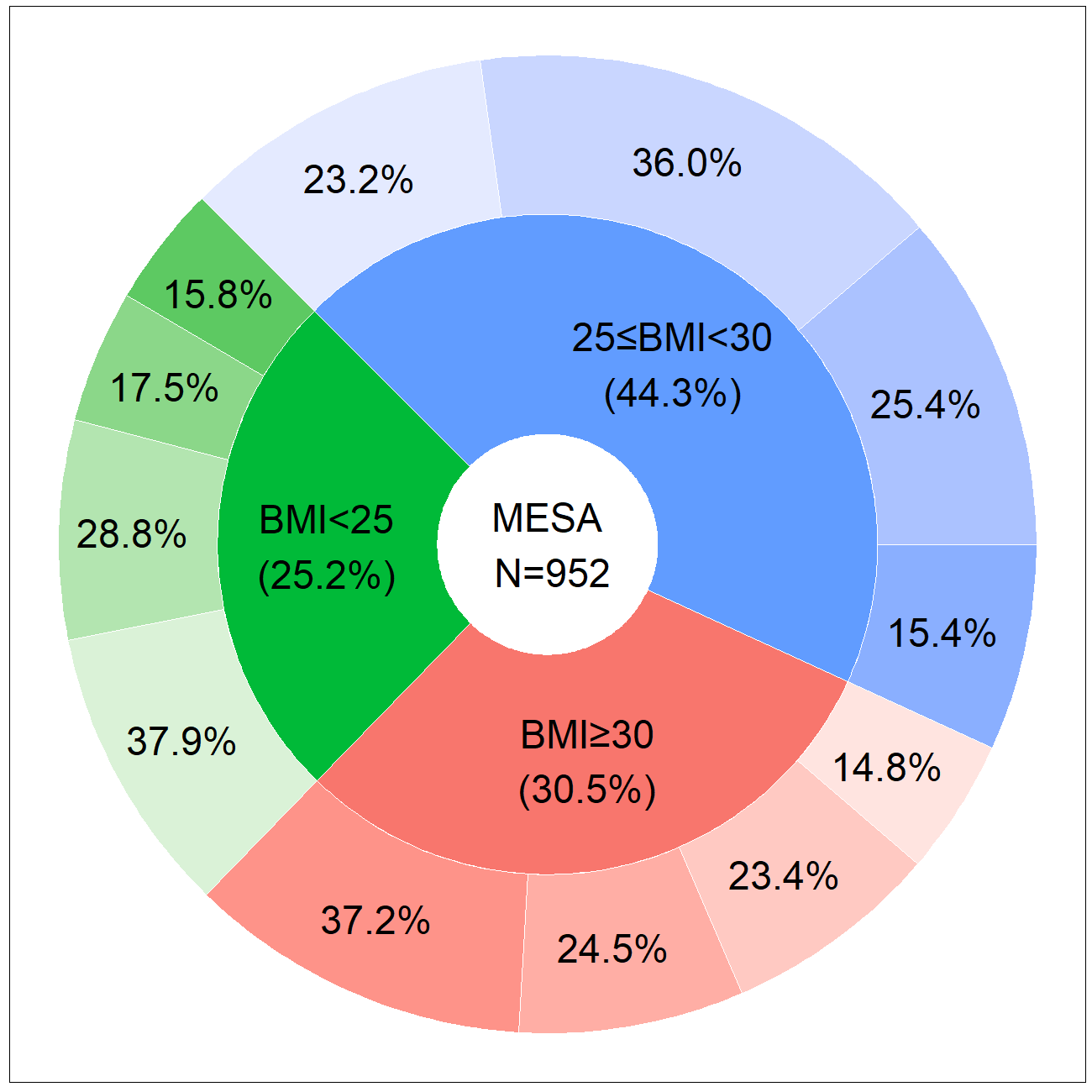

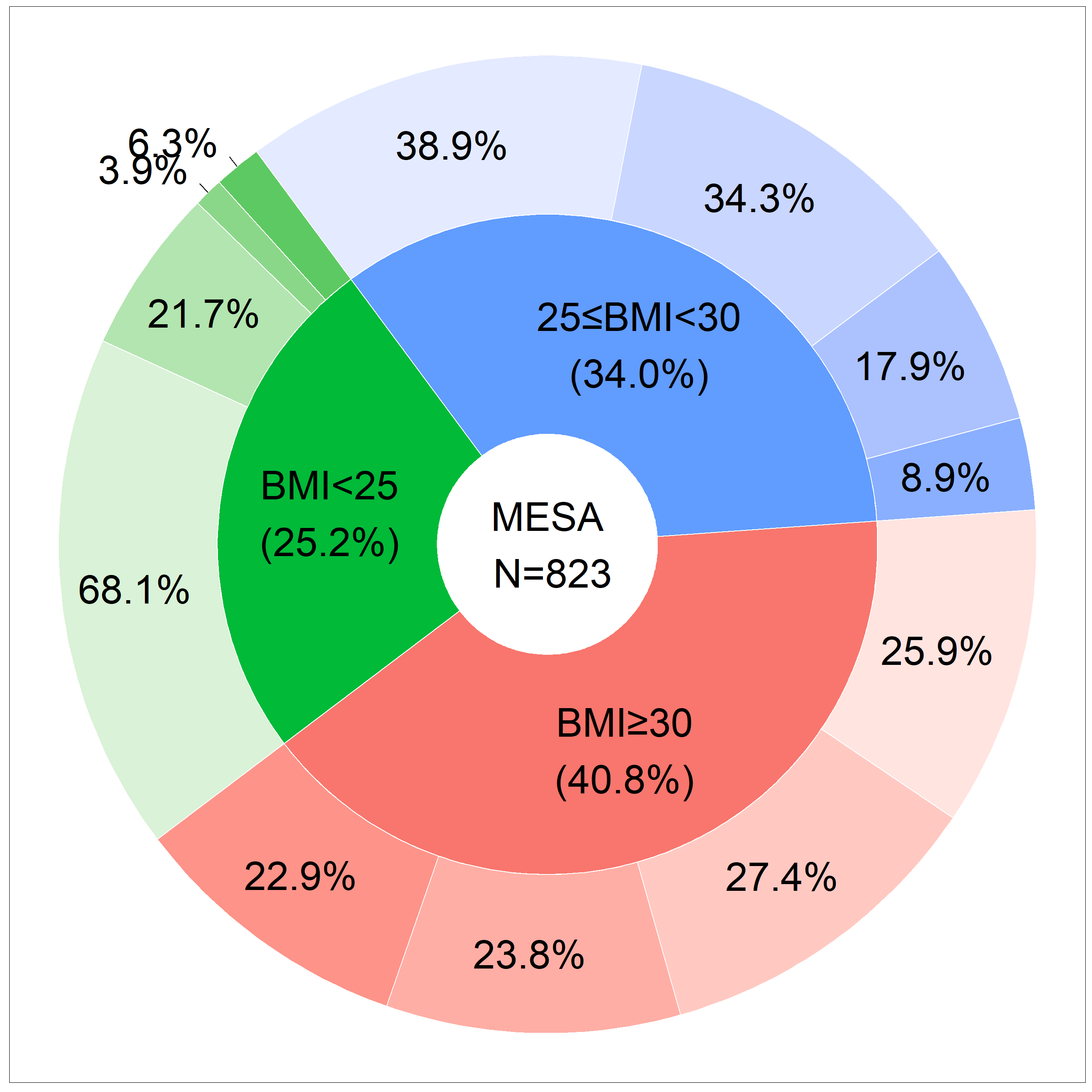

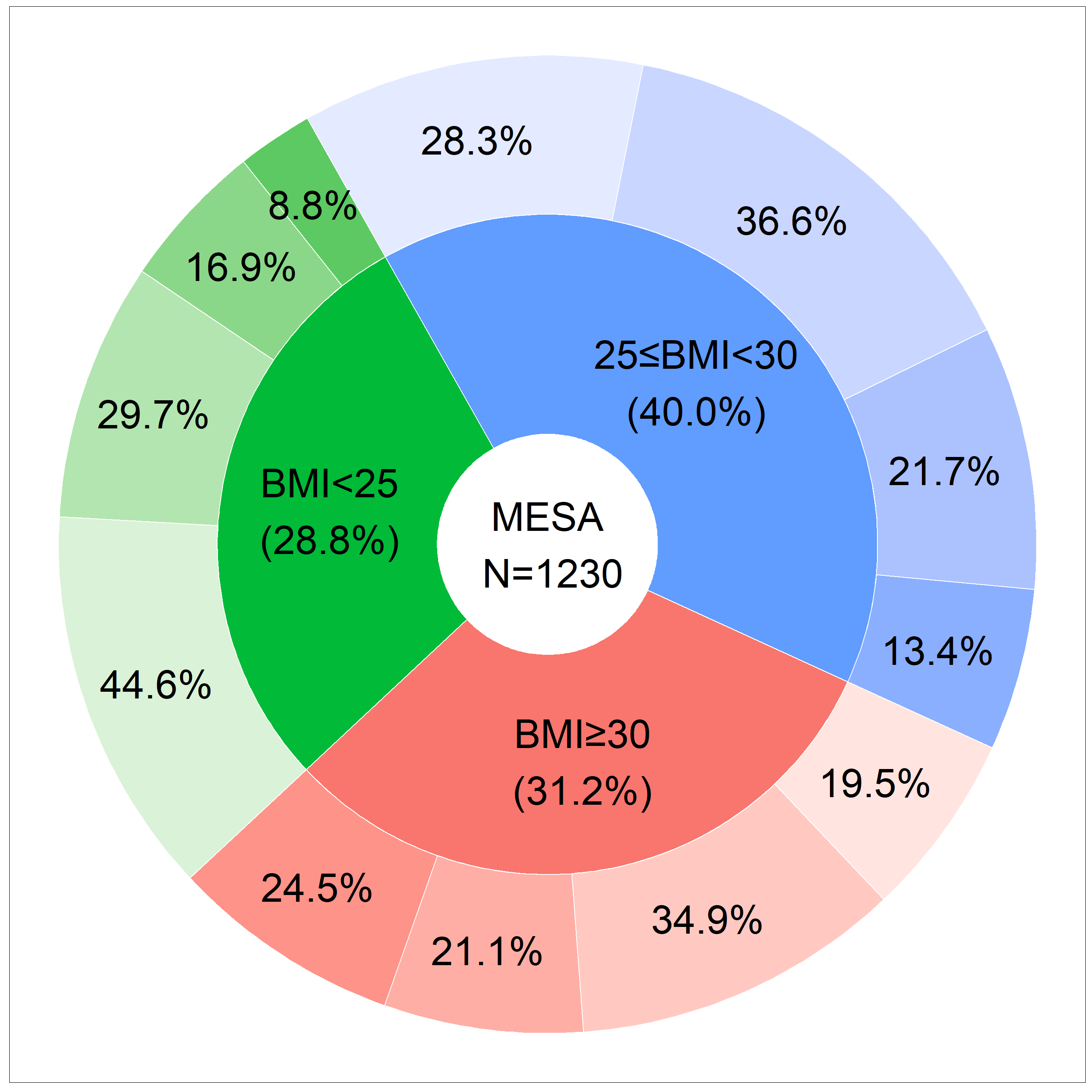


**Female Male**

**Age < 65 years Age ≥ 65 years**


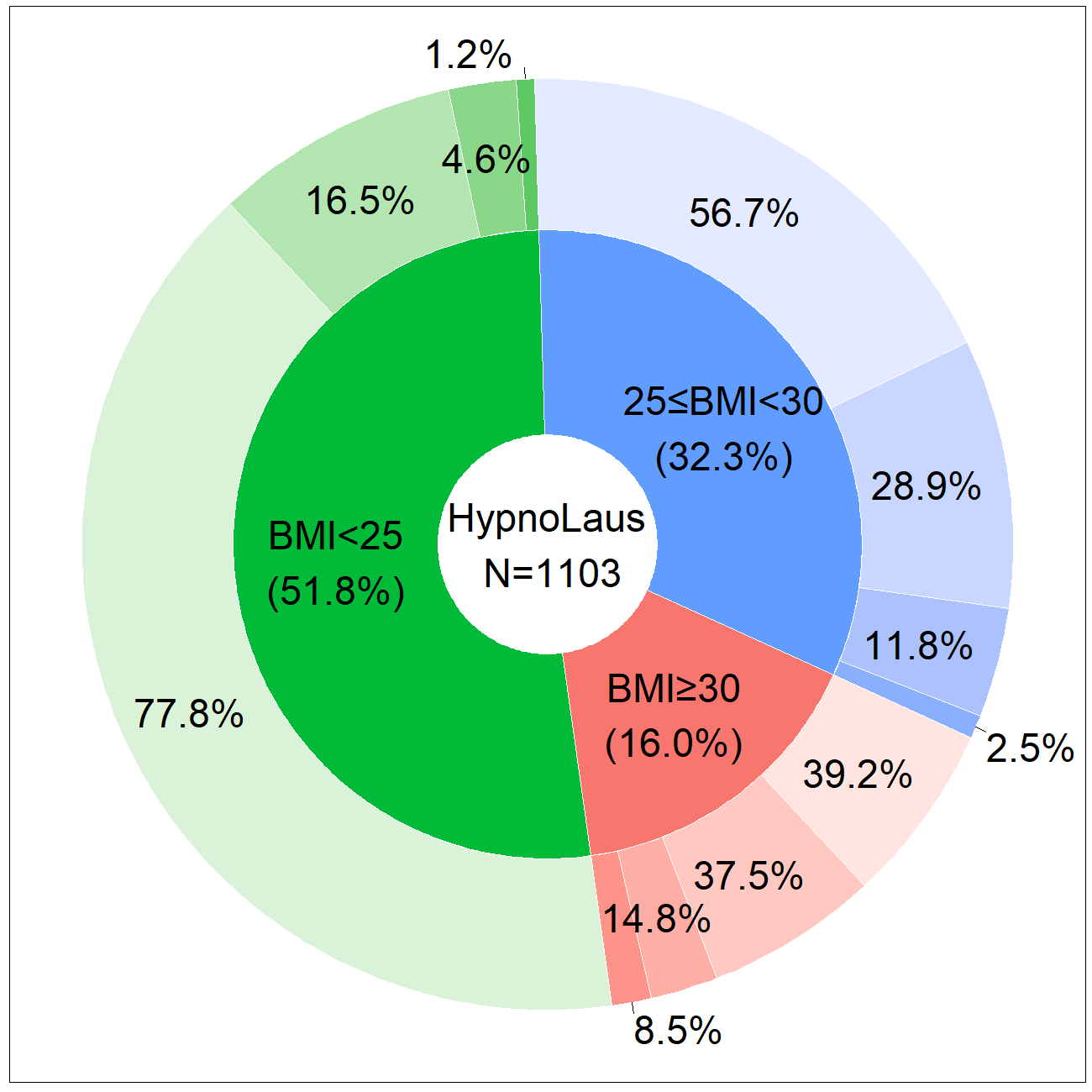

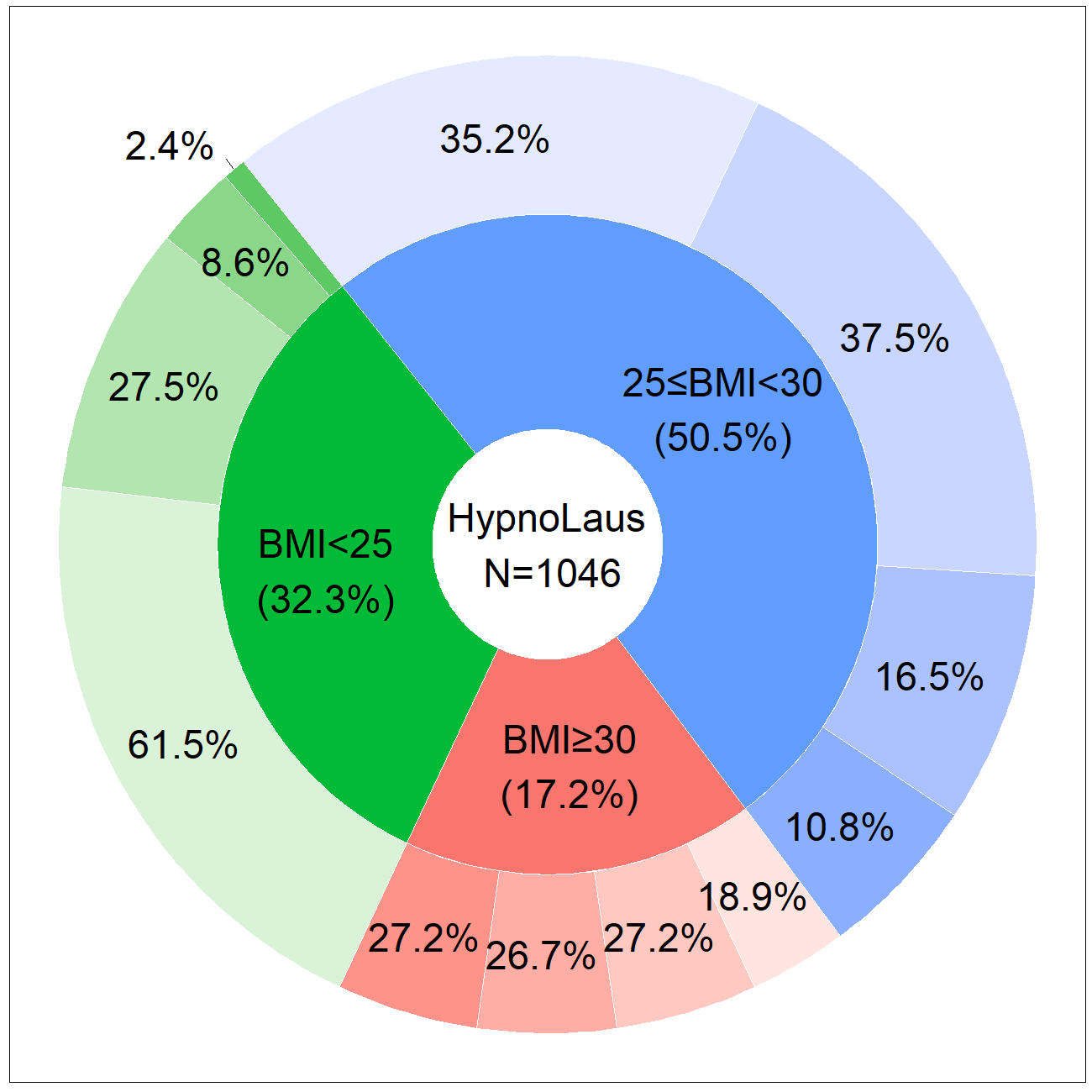

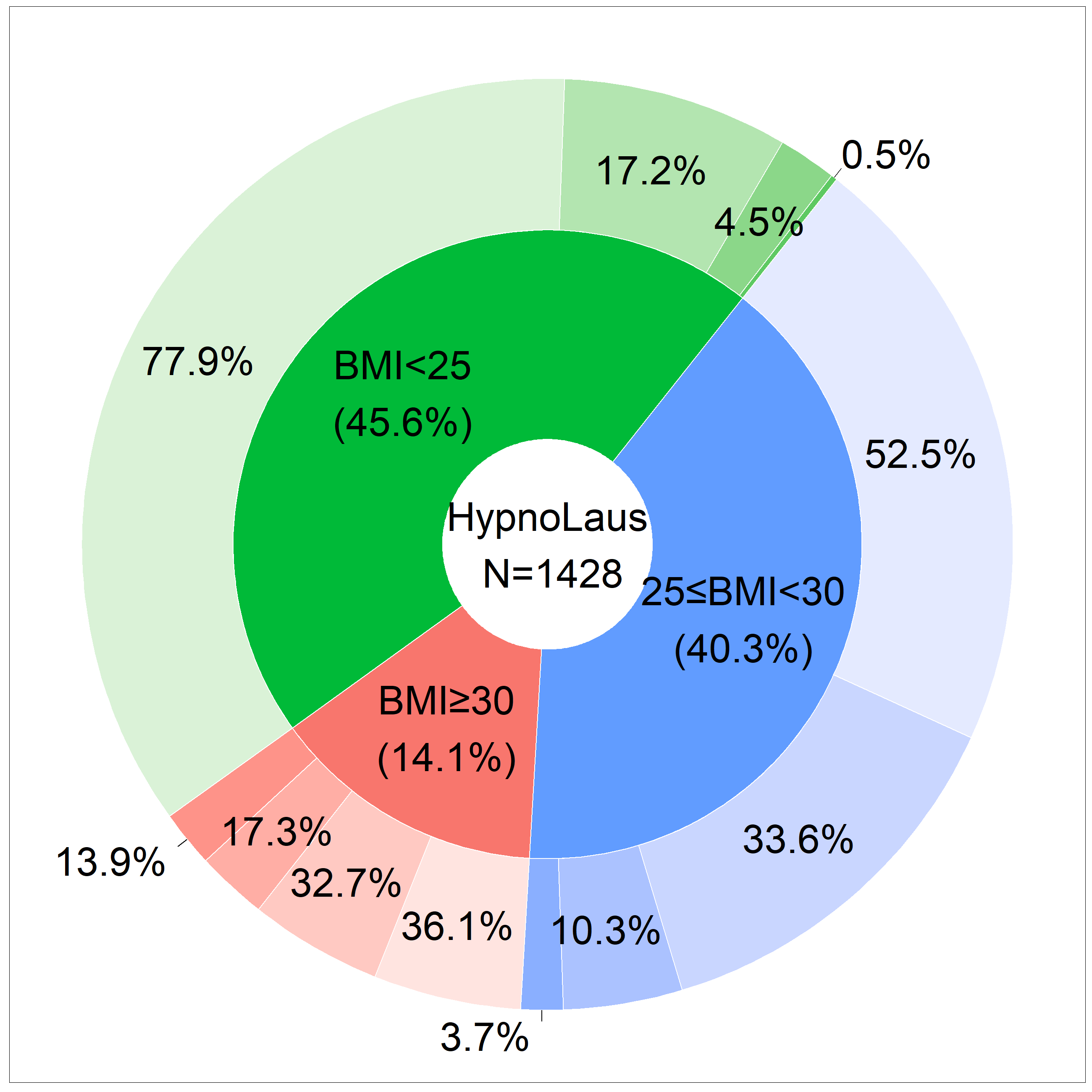

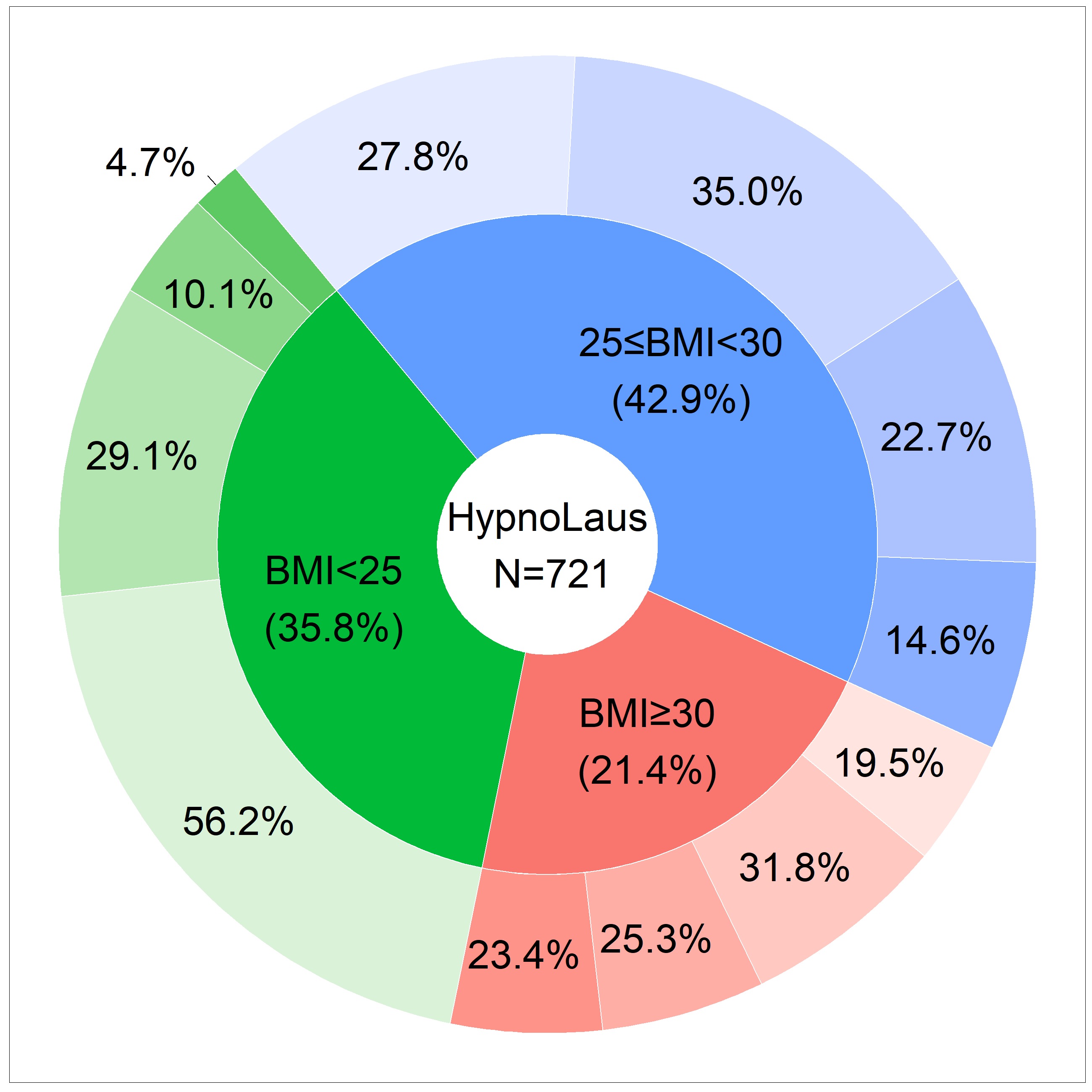


**eFigure 17. Pie charts to indicate the prevalence of different OSA severities in sex or age subgroups of weight groups in HypnoLaus study. OSA severities are shown from light to dark by AHI<5 events/h, 5≤AHI<15 events/h, 15≤AHI<30 events/h and AHI≥30 events/h. AHI: apnea-hypopnea index. BMI: body mass index (kg/m^2^). N: number.**
